# Supplementary material for: Program evaluation of a school-based mental health and wellness curriculum featuring yoga and mindfulness
Source: PLoS One. 2024 Apr 4;19(4):e0301028. doi: 10.1371/journal.pone.0301028 (PMC10994323; doi:10.1371/journal.pone.0301028)
Supplement: S1 File — (PDF) [file pone.0301028.s001.pdf]

Title : The Health and Wellness Curriculum Assessment
Approval Period: 04/24/2015 - 09/23/2015

Modification Form 3
Personnel Info 3
Participant Population 7
Study Location 8
General Checklist 8
Funding 10
Resources 11
Purpose, Study Procedures 12
Radioisotopes or Radiation Machines 23
Drugs,Devices,Reagents 23
Medical Equipment for Human Subjects and Laboratory Animals 25
Participant Population(a-g) 25
Participant Population(h-m) 26
Risks(a-e) 28
Risks(f) 31
Privacy And Confidentiality 32
Conflict Of Interest 32
Consent Background 35
Assent Background 41
HIPAA Background 43
Attachments 44

Title : The Health and Wellness Curriculum Assessment
Approval Period: 04/24/2015 - 09/23/2015

Obligations 46

Title : The Health and Wellness Curriculum Assessment
Approval Period: 04/24/2015 - 09/23/2015

Modification

1. Summarize your proposed changes.

1. Updated study procedures section a (4th paragraph) to reflect additional procedures for control group: "CONTROL SCHOOL PROCEDURES: Parents and caregivers of 3rd and 5th grade students (approximately 50) from Orchard School District in San Jose will receive a letter providing information about the study and requesting contact information for follow-up recruitment efforts. Research staff will contact interested parents for enrollment. Parents may also be invited to information sessions held at the school. Parents will be fully informed about the study and signatures will be obtained. Students from our control school will not be participating in the Ravenswood City School District Health and Wellness curriculum. All other procedures, questionnaires and assessments will be identical to the intervention schools, including feedback to parents regarding their child's intellectual functioning, sleep habits, etc."

2. Attached control group consent form (English and Spanish)

3. Attached copy of email serving as tentative agreement with control school district (pending signed MOU, which will be submitted asap).

2. Indicate Level of Risk

No Change

3. Update the Conflict of Interest (COI) section if any changes in COI have been made since the last protocol submission.

N Is there a change in the conflicting interest status for any existing personnel on this protocol?

Protocol Director

**Name**		**Degree (Program/year if student)**		**Position, e.g. Assistant Professor, Resident, etc.**
Victor G. Carrion		M.D.		Professor-Med Ctr Line
**Department** Psychiatry and Behavioral Sciences - Child and Adolescent Psychiatry and Child Development	5719	**Phone** [redacted]	[redacted]	**E-mail** [redacted]
**CITI Training current**				Y

Admin Contact

**Name**		**Degree (Program/year if student)**		**Position, e.g. Assistant Professor, Resident, etc.**
Travis Bradley		MPP		Research Coordinator
**Department** Psychiatry and Behavioral Sciences - Child and Adolescent Psychiatry and Child Development	5797	**Phone** [redacted]		**E-mail** [redacted]
**CITI Training current**				Y

Investigator

Title : The Health and Wellness Curriculum Assessment
Approval Period: 04/24/2015 - 09/23/2015

**Name**		**Degree** (**Program/year if student**)		**Position, e.g. Assistant Professor, Resident, etc.**
Vinod Menon		PhD		Professor
**Department** Psychiatry and Behavioral Sciences - Child and Adolescent Psychiatry and Child Development	5778	**Phone** [redacted]		**E-mail** [redacted]
**CITI Training current**				Y

Other Contact

**Name**		**Degree** (**Program/year if student**)		**Position, e.g. Assistant Professor, Resident, etc.**
Ruth O'Hara		PhD		Associate Professor
**Department** Psychiatry and Behavioral Sciences	5550	**Phone** [redacted]		**E-mail** [redacted]
**CITI Training current**				Y

Academic Sponsor

**Name**		**Degree** (**Program/year if student**)		**Position, e.g. Assistant Professor, Resident, etc.**
**Department**		**Phone**		**E-mail**
**CITI Training current**				

Other Personnel

**Name**		**Degree** (**Program/year if student**)		**Position, e.g. Assistant Professor, Resident, etc.**
John P Rettger		PhD		Clinical Researcher
**Department** Psychiatry and Behavioral Sciences - Child and Adolescent Psychiatry and Child Development	5719	**Phone** [redacted]	[redacted]	**E-mail** [redacted]
**CITI Training current**				Y

**Name**		**Degree** (**Program/year if student**)		**Position, e.g. Assistant Professor, Resident, etc.**
Veronica Alvarez		MSW		Community Based Research Assistant
**Department** Psychiatry and Behavioral Sciences - Child and Adolescent Psychiatry and Child	5719	**Phone**		**E-mail** [redacted]

Development				
**CITI Training current**				Y

**Name**<br>Ryan Matlow		**Degree** (**Program/year if student**)		**Position, e.g. Assistant Professor, Resident, etc.**<br>Instructor
**Department**<br>Psych/Major Laboratories and Clinical & Translational Neurosciences Incubator	5717	**Phone**		**E-mail**<br>[redacted]
**CITI Training current**				Y

**Name**<br>Shaozheng Qin		**Degree** (**Program/year if student**)		**Position, e.g. Assistant Professor, Resident, etc.**<br>J1 Exchange Visitor
**Department**<br>Psychiatry and Behavioral Sciences		**Phone**		**E-mail**<br>[redacted]
**CITI Training current**				Y

**Name**<br>Weidong Cai		**Degree** (**Program/year if student**)		**Position, e.g. Assistant Professor, Resident, etc.**
**Department**		**Phone**		**E-mail**<br>[redacted]
**CITI Training current**				Y

**Name**<br>Sarah Nicole Bostan		**Degree** (**Program/year if student**)		**Position, e.g. Assistant Professor, Resident, etc.**<br>Assessor
**Department**<br>Psychiatry and Behavioral Sciences - Child and Adolescent Psychiatry and Child Development	5590	**Phone**		**E-mail**<br>[redacted]
**CITI Training current**				Y

**Name**<br>Isabelle Cotto		**Degree** (**Program/year if student**)		**Position, e.g. Assistant Professor, Resident, etc.**<br>Social Science Research Assistant
**Department**<br>Psychiatry and Behavioral Sciences	5717	**Phone**		**E-mail**<br>[redacted]
**CITI Training current**				Y

Title : The Health and Wellness Curriculum Assessment
Approval Period: 04/24/2015 - 09/23/2015

**Name**<br>Alexander Basile		**Degree** (**Program/year if student**)		**Position, e.g. Assistant Professor, Resident, etc.**<br>Social Science Research Assistant
**Department**<br>Psychiatry and Behavioral Sciences - Child and Adolescent Psychiatry and Child Development	5719	**Phone**<br>[redacted]		**E-mail**<br>[redacted]
**CITI Training current**				Y
**Name**<br>Mr Keith Daniel Sudheimer		**Degree** (**Program/year if student**)		**Position, e.g. Assistant Professor, Resident, etc.**<br>Assessor
**Department**<br>Psychiatry and Behavioral Sciences	5717	**Phone**<br>[redacted]		**E-mail**<br>[redacted]
**CITI Training current**				Y
**Name**<br>Lauren Ashley Anker		**Degree** (**Program/year if student**)		**Position, e.g. Assistant Professor, Resident, etc.**<br>Research Assistant
**Department**<br>Psychiatry and Behavioral Sciences	5717	**Phone**<br>[redacted]		**E-mail**<br>[redacted]
**CITI Training current**				Y
**Name**<br>Jennifer Ka Yu Lai		**Degree** (**Program/year if student**)		**Position, e.g. Assistant Professor, Resident, etc.**
**Department**<br>Psychiatry VA Research		**Phone**		**E-mail**<br>[redacted]
**CITI Training current**				Y
**Name**<br>Joshua Z Tal		**Degree** (**Program/year if student**)		**Position, e.g. Assistant Professor, Resident, etc.**<br>Data Assistant
**Department**<br>Psychiatry and Behavioral Sciences	5717	**Phone**		**E-mail**<br>[redacted]
**CITI Training current**				Y
**Name**<br>Katherine V Espana		**Degree** (**Program/year if student**)		**Position, e.g. Assistant Professor, Resident, etc.**<br>Social Science Research Assistant
**Department**<br>Psychiatry and	5797	**Phone**		**E-mail**<br>[redacted]

Title : The Health and Wellness Curriculum Assessment
Approval Period: 04/24/2015 - 09/23/2015

Behavioral Sciences - Child and Adolescent Psychiatry and Child Development				
**CITI Training current**				Y

**Name** Jairo Carretero		**Degree** (**Program/year if student**)		**Position, e.g. Assistant Professor, Resident, etc.** Social Science Research Assistant
**Department** Psychiatry and Behavioral Sciences - Child and Adolescent Psychiatry and Child Development	5717	**Phone**		**E-mail** [redacted]
**CITI Training current**				Y

**Name** Sophia Schoenberg		**Degree** (**Program/year if student**)		**Position, e.g. Assistant Professor, Resident, etc.** Social Science Research Assistant
**Department**		**Phone**		**E-mail** [redacted]
**CITI Training current**				Y

**Name** Kristina Mendez		**Degree** (**Program/year if student**)		**Position, e.g. Assistant Professor, Resident, etc.** Research Assistant
**Department**		**Phone**		**E-mail** [redacted]
**CITI Training current**				Y

**Name** Sophie Borst		**Degree** (**Program/year if student**)		**Position, e.g. Assistant Professor, Resident, etc.**
**Department**		**Phone**		**E-mail** [redacted]
**CITI Training current**				Y

Participant Population(s) Checklist — Yes/No

Population	Yes/No
• Children (under 18)	Y
• Pregnant Women and Fetuses	N
• Neonates (0 - 28 days)	N
• Abortuses	N
• Impaired Decision Making Capacity	N
• Cancer Subjects	N

- Laboratory Personnel N
- Healthy Volunteers Y
- Students Y
- Employees N
- Prisoners N
- Other (i.e., any population that is not specified above) N
- International Participants

  Please enter the countries separated by comma

Study Location(s) Checklist Yes/No

- Stanford University Y
- Clinical & Translational Research Unit (CTRU)
- Stanford Hospital and Clinics
- Lucile Packard Children's Hospital (LPCH)
- VAPAHCS (Specify PI at VA)
- Other (Click ADD to specify details) Y

Location / Country	Location	Contact Name	Contact Phone	Contact Email	Permission ?	Engaged?
Ravenswood City School District	US	Lorena Morales-Ellis	[redacted]		Y	N

General Checklist

Multi-site Yes/No

- Is this a multi-site study? A multi-site study is generally a study that involves one or more medical or research institutions in which one site takes a lead role.(e.g., multi-site clinical trial) N

Collaborating Institution(s) Yes/No

- Are there any collaborating institution(s)? A collaborating institution is generally an institution that collaborates equally on a research endeavor with one or more institutions. Y

Institution Name	Contact Name	Contact Phone	Contact Email	Permission?	Engaged?
The Sonima Foundation	Carmen Blanco		[redacted]	Y	N
University of San Diego	Scott Himelstein		[redacted]	Y	Y

Title : The Health and Wellness Curriculum Assessment
Approval Period: 04/24/2015 - 09/23/2015

**Cancer Institute**	**Yes/No**
• Cancer-Related Studies (studies with cancer endpoints), Cancer Subjects (e.g., clinical trials, behavior/prevention) or Cancer Specimens (e.g., blood, tissue, cells, body fluids with a scientific hypothesis stated in the protocol).	N

**Clinical Trials**	**Yes/No**
• Investigational drugs, biologics, reagents, or chemicals?	N
• Commercially available drugs, reagents, or other chemicals administered to subjects (even if they are not being studied)?	N
• Investigational Device / Commercial Device used off-label?	Y
• IDE Exempt Device (Commercial Device used according to label, Investigational In Vitro Device or Assay, or Consumer Preference/Modifications/Combinations of Approved Devices)	Y
• Will this study be registered on??? clinicaltrials.gov? (See Stanford decision tree)	N
• Who will register for ClinicalTrials.gov?<br>NCT#	

**Tissues and Specimens**	**Yes/No**
• Human blood, cells, tissues, or body fluids (tissues)?	Y
• Tissues to be stored for future research projects?	N
• Tissues to be sent out of this institution as part of a research agreement? For guidelines, please see https://sites.stanford.edu/ico/mtas https://sites.stanford.edu/ico/mtas	Y

**Biosafety** (**APB**)	**Yes/No**
• Are you submitting a Human Gene Transfer investigation using a biological agent or recombinant DNA vector? If yes, please complete the Gene Transfer Protocol Application Supplemental Questions and upload in Attachments section.	N
• Are you submitting a Human study using biohazardous/infectious agents? If yes, refer to the Administrative Panel on BioSafety website prior to performing studies.	N
• Are you submitting a Human study using samples from subjects that are known or likely to contain biohazardous/infectious agents? If yes, refer to the Administrative Panel on BioSafety website prior to performing studies.	N

**Human Embryos or Stem Cells**	**Yes/No**
• Human Embryos or Gametes?	N
• Human Stem Cells (including hESC, iPSC, cancer stem cells, progenitor cells)	N

**Veterans Affairs (VA)**	**Yes/No**
• The research recruits participants at the Veterans Affairs Palo Alto Health Care System(VAPAHCS).	N
• The research involves the use of VAPAHCS non-public information to identify or contact human research participants or prospective subjects or to use such data for research purposes.	N
• The research is sponsored (i.e., funded) by VAPAHCS.	N
• The research is conducted by or under the direction of any employee or agent of VAPAHCS (full-time, part-time, intermittent, consultant, without compensation (WOC), on-station fee-basis, on-station contract, or on-station sharing agreement basis) in connection with her/his VAPAHCS responsibilities.	N
• The research is conducted using any property or facility of VAPAHCS.	N

**Equipment**	**Yes/No**
• Use of Patient related equipment? If Yes, equipment must meet the standards established by Biomedical Engineering (BME) (650-725-5000)	Y
• Medical equipment used for human patients/subjects also used on animals?	Y
• Radioisotopes/radiation-producing machines, even if standard of care? ; More Info	N

**Payment**	**Yes/No**
• Subjects will be paid/reimbursed for participation? See payment considerations.	Y

**Funding**	**Yes/No**
• Training Grant?	N
• Program Project Grant?	Y
• Federally Sponsored Project?	N
• Industry Sponsored Clinical Trial?	N

Funding

Funding - Grants/Contracts

Funding Administered By : STANFORD

SPO # (if available) :

Grant # (if available) :

Funded By (include pending) : The Sonima Foundation

Principal Investigator : Victor G. Carrion. M.D.

Grant/Contract Title if different from Protocol Title :

N For Federal projects, are contents of this protocol consistent with the Federal proposal?

Title : The Health and Wellness Curriculum Assessment
Approval Period: 04/24/2015 - 09/23/2015

N	Is this a Multiple Project Protocol (MPP)?
N	Is this protocol under a MPP?

Funding - Fellowships

Gift Funding

Dept. Funding

Other Funding

Resources :

a) Qualified staff.

Please state and justify the number and qualifications of your study staff.

The team's Principal Investigator (PI): Victor G. Carrion, M.D. and Co-PI's Vinod Menon, Ph.D. (Professor and Director of the Stanford Cognitive and Systems Neuroscience Lab) and Ruth O'Hara, Ph.D. (Associate Professor and Faculty at the Stanford Sleep Lab) have significant expertise and publication histories in the proposed methods. Their NIH and private foundation funded studies support laboratories that include Post-doctoral Fellows, Technicians, Clinicians and Research Assistants. The group has a history of collaboration and expertise working with children and vulnerable populations.

Dr. Carl Weems is methods and statistics consultant on this project. He is NOT involved in data collection or work with PHI

b) Training.

Describe the training you will provide to ensure that all persons assisting with the research are informed about the protocol and their research-related duties and functions.

All staff have completed all necessary ethical and human research training including HIPAA regulations. Regular weekly meetings will be held with research staff to supervise the process and ensure compliance with protocols.

c) Facilities.

Provide the location(s) **where the research will be conducted, including physical address if not conducted on site at Stanford University, Stanford Hospital on Pasteur Dr., Lucile Packard Children's Hospital on Welch Rd. or VAPAHCS. Describe the facilities and resources available to conduct the research at these sites.**

Trained instructors will provide the intervention and assessments in elementary and middle school classrooms at seven public schools in the Ravenswood City School District as part of the district's curriculum.

Neuroimaging data will be acquired at Stanford's Richard M Lucas Center for Imaging.

Salivary cortisol samples will be collected by participants parents in their homes and sent by Stanford research staff to the Salimetrics Saliva Lab on Friday afternoons.

Ambulatory Polysomnography assessments will be conducted by trained research staff in the home of the participant. Data will be analyzed at the Late Life and Lifespan Approaches to Neuropsychiatric Disorders Lab at the Stanford School of Medicine.

The Early Life Stress and Pediatric Anxiety Program has lab space located in the Department of Psychiatry and Behavioral Sciences and will use this space for email communication and supervisory meetings with study staff. Data related to the study will be stored in a locked cabinet in the Department of Psychiatry and Behavioral Science. Telecommunication services, laser quality printers, and faxing facilities are also available.

d) Sufficient time.

Explain the time that you and your research team will allocate to perform the research activities, including data analysis.

The Health and Wellness Curriculum Study is a four-year project. We will begin in September, 2014 starting with a six month period for implementation of procedures and baseline assessments followed by 12 month and 18 month assessments.

e) Access to target population.

Explain and justify whether you will have access to a population that will allow recruitment of the required number of participants.

The Ravenswood City School District is in partnership with the Early Life Stress and Pediatric Anxiety Program to recruit 800 3rd and 5th graders to participate in the study, all of whom will be receiving the new curriculum.

f) Access to resources if needed as a consequence of the research.

State whether you have medical or psychological resources available that participants might require as a consequence of the research when applicable. Please describe these resources.

This is a minimal risk study. Participants can be referred to the Stanford Clinic as well as the Ravenswood Family Health Center should they feel the need for counseling services in response to their participation.

Additionally, members of our research team include a licensed clinical psychologist and a licensed psychiatrist capable of providing direct care to participants that experience unlikely adverse reactions to interventions and assessments.

g) Lead Investigator or Coordinating Institution in Multi-site Study.

Please explain (i) your role in coordinating the studies, (ii) procedures for routine communication with other sites, (iii) documentation of routine communications with other sites, (iv) planned management of communication of adverse outcomes, unexpected problems involving risk to participants or others, protocol modifications or interim findings.

1. Purpose

a) In layperson's language state the purpose of the study in 3-5 sentences.

The overarching goal of our study is to examine the effects of a new Health and
Wellness
Curriculum which is now being implemented in the Ravenswood City School
District in East
Palo Alto. The Health and Wellness Curriculum, which was developed by the

Sonima
Foundation, includes exercise based on yoga, common exercise regimes, relaxation
techniques, mindfulness practices, and nutrition education, and is being introduced this year
to the Ravenswood City Schools, independent of our research. The new curriculum is expected
to yield dramatic positive effects on students' socio-emotional development, which we aim to
examine through this study.

This project will be the first systematic neuroscience study of skill-building in children via a
multi-method assessment that includes structural MRI, functional MRI, salivary cortisol and
sleep architecture data. These are methods that have proven critical in the fundamental
understanding of stress in children. Our research will identify critical biopsychosocial
components responsible for cognitive, behavioral, emotional and academic improvement,
effective implementation, and the sustainability of the program.

The Sonima Foundation's K-12 Health and Wellness curriculum was first implemented in the
Encinitas Union School District in Encinitas, CA, and and is currently being implemented and
several dozen schools in California, New York, Florida and Texas. The curriculum has been
reviewed, commented on and in some cases edited, by doctors, scientists and researchers at
the University of San Diego, Duke University, and Long Island University. The role of the
Sonima Foundation in the current study is that of a funding partner, and and will not involve
any identifiable documents, data or specimens. Sonima staff will not have direct contact with
participants in a research capacity. Funding for this research from Sonima is not a fee for
service. All research activities including research questions, study design, data collection and
analysis will be independently controlled by the Stanford research team.

b) State what the Investigator(s) **hope to learn from the study. Include an assessment of the importance of this new knowledge.**

Assessments will include Cognitive, Academic, Neurobehavioral, Sleep, Endocrinological
and Neuroimaging modalities; (the CAN SEN assessments). These components are essential to
our understanding of the fundamental mechanisms of stress, adversity, resilience, healing and
recovery.

We theorize that our assessments will demonstrate that the Health and Wellness Curriculum

will effect regions of interest such as the insula, anterior cingulate, amygdala,hippocampus and pre-frontal cortex and improve their function. These regions play vital roles in the ability to perceive and react to cognitive and emotional information. We postulate that regulation of sleep and activity of the limbic-hypothalamic-pituitary-adrenal axis are important mechanisms by which effective interventions can improve brain function, cognition and behavior. The knowledge base that will result from these studies will inform the implementation and underlying biology of the curriculum and will provide a rationale for further development and dissemination.

c) **Explain why human subjects must be used for this project.** (**i.e. purpose of study is to test efficacy of investigational device in individuals with specific condition; purpose of study is to examine specific behavioral traits in humans in classroom or other environment**)

The purpose of this study is to examine the improved socioemotional development of children enduring a variety of adverse living conditions and ongoing traumatic stressors after receiving a new curriculum designed to help them cope with such adversities.

2. Study Procedures

a) **Please SUMMARIZE the research procedures, screening through closeout, which the research participant will undergo. Sections in the protocol attached in section 16 can be referenced, BUT do not copy the clinical protocol. Be clear on what is to be done for research and what is part of standard of care. For research involving collaborators, please specify the respective roles of Stanford and each collaborator on the protocol.**

Parents and caregivers of Ravenswood students in the 3rd and 5th grades (n=800) will receive a letter providing informed consent and describing our protocol for the questionnaire portion of the study that will take place during school. The District will also call participants notifying them of the study. Parents will be provided with study contact information in order to ask any questions and address any concerns they may have about the study protocol. Parents will be fully informed about the study, but a signature on the consent form will not be required. We request a Waiver of Documentation of Consent because it is extremely difficult to secure a signature from parents in this population as many work more than one job, family life can be very unpredictable and many parents are not actively involved in school activities. If a parent wishes to have his or her child opt out of the study, they may contact research study staff to notify them. Research study staff will follow up with as many parents as possible to complete the additional questionnaires (like Medical and Developmental History

Form)
covered through informed consent without signature. The original Questionnaire Consent will
still be used to secure signatures from parents when possible, because we will need signed
consent to access their child's academic records and to comply with FERPA. All students
participating in the Questionnaire (and other) components of the study will still be asked to
sign assent forms.

Parents will also be invited to information sessions held by school and
research staff at schools participating in the study. Caregivers will be educated in the
assessment process and asked to indicate the additional assessment components that they
and their children would be interested in completing. Investigators will present all
subjects and their caregivers with copies of the Stanford University Internal Review Board
(IRB)-approved informed consent and assent forms for the more comprehensive consent form
(which includes cognitive testing, sleep, cortisol and neuroimaging portions of the study) and
a signed consent form will be obtained for these additional components.

Trained instructors will provide the Health and Wellness Curriculum at the Ravenswood City
schools. Each school receiving the curriculum will have a dedicated space and receive a 30
minute class twice a week. Through group discussion, nutrition and character education, and
breathing exercises, students will receive instruction to focus their attention, calm
their minds, reduce stress, and think before reacting.

CONTROL SCHOOL PROCEDURES: Parents and caregivers of 3rd and 5th grade students (approximately 50) from Orchard School District in San Jose will receive a letter providing information about the study and requesting contact information for follow-up recruitment efforts. Research staff will contact interested parents for enrollment. Parents may also be invited to information sessions held at the school. Parents will be fully informed about the study and signatures will be obtained. Students from our control school will not be participating in the Ravenswood City School District Health and Wellness curriculum. All other procedures, questionnaires and assessments will be identical to the intervention schools, including feedback to parents regarding their child's intellectual functioning, sleep habits, etc.

At three separate points over the course of the next three years, all consenting participants
will receive an academic and neurobheavioral assessment via paper questionnaires in the
classroom. The research staff will measure children's academic functioning abilities, as well as
moods, thoughts and behavioral symptoms, which will be employed as a control

variable in
the proposed analyses. This will be accomplished by administering the Wide Range
Achievement Test (WRAT-IV), the Behavior Assessment System for Children (BASC-II), the
Responses to Stress Questionnaire, and the Behavior Rating Inventory of Executive Function
(BRIEF).

The BASC-2 provides an integrated and comprehensive assessment of the child's adaptive and
behavioral strengths and weaknesses. The BRIEF will be utilized to collect caregiver and
teacher ratings to assess executive functioning in the home and school environments.
The BASC has a parent form as well, and the BRIEF has both parent and teacher forms that will
be completed to supplement self report data from students. All of these assessments are
attached in Section 16. In addition, academic performance, attendance, number of
suspensions, expulsions and disciplinary issues will be available in each student's School Wide
Information System (SWIS) record, facilitated by the Ravenswood City School District. The
WRAT-IV will be used to assess participants' academic functioning, including arithmetic,
reading, and spelling.

At Ravenswood, at least 100 students from the original 800 student sample will be recruited for the
cognitive
(plus additional questionnaires), sleep, and endocrinology portions of the study. From this
100 student sub sample, at least 30 students will be recruited to participate in the
neuroimagining component of the study.

Cognitive and IQ abilities will be assessed using the Kaufman Assessment Battery for Children
2 (K-ABC II): all scales and subtests. During the cognitive testing portion of the study,
participants will also complete the reading section of the Wide Range
Achievement Test (WRAT-IV) and the Trauma Symptoms Checklist for Children
(TSCC).

The control group will be recruited at a later date from neighboring schools. A modification to
the current protocol will be submitted before any research will be conducted involving said
control group. This modification will include a unique consent form as well as updated
procedures, specific to the control group.

For the salivary cortisol collection, families will receive instruction and materials from research

staff to perform the collection in their own homes on a weekend that is convenient for the
family. Diurnal salivary cortisol will be collected by parents using the Salimetrics Children's
Swab for 2 consecutive days at five specific times: within 20 minutes of waking up, before
breakfast, before lunch, before dinner and before bedtime. Results from these time periods
will serve as an index of baseline cortisol rhythmicity. This process will occur a total of three
times (once at each assessment time).

The families of students identified as eligible to participate in the neuroimaging component of
the study will be contacted by research staff to schedule an appointment at the Stanford Lucas
Imaging Center. Children will complete the Tanner stages form to assess their scale of
physical development (puberty) and handedness form to determine if they are right or left
handed and Domain-Specific Impulsivity Scale for Children (DSIS-C) questionnaire, which
measures impulsivity traits in the domains of schoolwork and interpersonal relationships, to
be used in concert with neuroimaging data. fMRI, DTI and sMRI data will be acquired on a 3T
scanner with a state-of-the-art 32-channel parallel imaging coil.

Children will perform up to four cognitive and emotional experiments during fMRI scanning.
A stop signal task will be used to investigate brain plasticity in systems associated with
attention and cognitive control. In stop-signal task, participants will make button presses in
response to left/right-pointing arrows and occasionally cancel their prepared responses.
A balloon analogue risk task (BART) will be used to investigate brain plasticity in systems
associated with risk-taking and risk avoidance. In BART, participants choose between
pumping a balloon or to cash out on each trial. Every successful pump adds $0.25 to their
reward on that trial. If the balloon explodes, the accumulated earning on that trial will be lost.
Participants will be told that they will receive monetary reward for their grand total earnings
from the task, subject to a maximum of $15.

An emotional labeling task will be used to examine brain plasticity in systems associated with
emotion perception. Participants will decide which of two facial expressions presented on the
bottom of the screen match the facial expression at the top of the screen. The faces have
either angry or fearful expression.

Title : The Health and Wellness Curriculum Assessment
Approval Period: 04/24/2015 - 09/23/2015

An emotional regulation task will be used to examine brain plasticity in systems associated
emotional control. Participants will view 60 neutral and aversive images which are carefully
selected for pediatric populations based on previous studies. When participants see the
instructional cue "Look", they just naturally react to the picture. When participants see the cue
"Decrease" , they will have to tell themselves a story about the picture that make themselves
feel less negative (i.e., to reappraise). This reappraisal strategy has been shown to
successfully reduce negative emotion in children as young as 10 years-old. Prior to the fMRI
experiment, children will be given examples about how to reappraise (e.g., imagining it's just
a scene from a movie) and asked to report their reappraisals aloud during training to ensure
understanding of the instructions.

Data for each experiment will be acquired across 2 runs (except only 1 run for the emotional
labeling task), each lasting 6 minutes, in order to minimize fatigue while providing adequate
statistical power to detect effects of interest. This process will occur a total of three times
(once at each assessment time).

Participants will also complete a Delay Discounting Task outside of the MRI scanner. We will
use a computerized version of the Monetary Choice Questionnaire to measure delay
discounting. During each trial of the task, participants will be presented with a choice between
an immediate monetary reward ($11-80), and a larger reward ($25-85) delayed by 7-186
days. Participants will be told that they will receive 10% of the monetary reward from a
randomly selected trial. Participants will be paid at the end of the session if the chosen trial
was an immediate reward. For delayed rewards, participants will be mailed payment at the
delay specified in the trial.

Two salivary cortisol samples will be collected by researchers: one at beginning of the scan
and the other one after the scan. One salivary hormone sample will be collected at the end of
the scan. Samples will be stored in a fridge with temperature lower than -20?C and then sent
by Stanford research staff to the Salimetrics Saliva Lab on Friday afternoons.

To assess sleep, research staff will then schedule an initial visit in participants' homes to
explain in depth the sleep assessment which will be conducted overnight in the

Title : The Health and Wellness Curriculum Assessment
Approval Period: 04/24/2015 - 09/23/2015

home. Once they are satisfied with the answers to all of their questions regarding the study, we will begin the desensitization process (this can also be scheduled for another time if preferred) in order to prepare for the overnight ambulatory polysomnography (PSG) of sleep. A significant part of the preparation for the PSG involves working with participants and their parents using systematic desensitization in order to make them more comfortable with the PSG process. Systematic desensitization is a type of behavioral therapy, and will be conducted in our investigation by the Post-Doctoral fellow who will have a clinical psychology degree. In the process of systematic desensitization an individual learns to cope and overcome their anxiety in a sequence of steps by increasing exposure to any source of the anxiety. Participants will be gradually introduced to the PSG equipment over the course of a week.

When the family is ready, research staff will employ the Compumedics Safiro? ambulatory PSG monitoring system to assess sleep. This ambulatory device includes a cap and several electrodes, and records EEG, EMG, expiratory/inspiratory nasal airway pressure, nasal/oral airflow, finger pulse oximetry, electrocardiogram (ECG), movements of the rib cage and abdomen, snoring, body position and bilateral anterior tibialis EMG. Sensors will be placed and calibrated, and signal quality and impedance checked by a trained research technician in the home of the subject. The compumedics system uses a recording cap, with electrodes embedded in the cap, which minimizes the discomfort, and removes the risk of electrodes becoming dislodged during the night. All sensors will be plugged into the recording device, and Compumedics Safiro? recordings are started. The participant will then go to sleep in their own bed, and should not encounter any disturbances or discomfort.

Data and equipment will be retrieved the next day by research staff, and stored digitally and hand-scored according to standard definitions as defined by the American Academy of Sleep Medicine. The systems provides multiple sleep parameters in the course of a full PSG which we then have manually scored by an experienced registered PSG technician (RPSGT). Parents also complete the Child's Sleep Habits Questionnaire (CSHQ), which is the most frequently employed measure assessing the quality and type of sleep experienced by their child, and

which also assesses for daytime sleepiness. Parents also note the time at which the child goes to bed, the duration to fall asleep, time of lights out, number of awakenings, and time of rising.
This process will occur a total of three times (once at each assessment time).

b) Explain how the above research procedures are the least risky that can be performed consistent with sound research design.

There are few potential risks of having an MRI scan, as it is a non-invasive procedure and does not involve any radiation. The MRI scanner uses a strong magnetic field. Before the scan, participants will be asked to empty their pockets and remove any clothing containing metal such as a belt. Any piercings must also be removed. This is because the main potential hazard comes from loose metal objects, which, if taken near the scanner, could be dangerous. We will go through a screening questionnaire with participants before the scan to ensure there are no other reasons why they should not have a scan such as having had recent metal implants put in or having a pacemaker.

The evaluations and assessments used in this study may include questions that are personal that may make participants uncomfortable or upset. Some questions are of a sensitive nature.
Participants have the right to refuse to answer any individual questions.

The sleep equipment may be uncomfortable but the desensitization process is in place to minimize any discomfort. There is a minimal risk of skin irritation as a result of the gel applied to adhere the cap. Should this occur, it is within the participants rights and ability to immediately stop the procedure.

c) State if deception will be used. If so, provide the rationale and describe debriefing procedures. Since you will not be fully informing the participant in your consent process and form, complete an alteration of consent (**in section 13**)**. Submit a debriefing script** (**in section 16**).

No deception will be used.

d) State if audio or video recording will occur. Describe what will become of the recording after use, e.g., shown at scientific meetings, erased. Describe the final disposition of the recordings.

No audio of video recordings will occur.

e) Describe alternative procedures or courses of treatment, if any, that might be advantageous to the participant. Describe potential risks and benefits associated with these. Any standard treatment that is being withheld must be disclosed in the consent process and form. (**i.e. standard-of-care drug, different interventional procedure, no procedure or treatment, palliative care, other research studies**).

Because this is a naturalistic study, it does not exclude any of the children from receiving any available treatments outside the program.

The alternative to participating in this study is not to participate.

f) Will it be possible to continue the more (most) appropriate therapy for the participant(s) after the conclusion of the study?

Participants will be educated on a variety of therapeutic practices that they will be able to continue on their own after the study has reached it's endpoint.

g) Study Endpoint. What are the guidelines or end points by which you can evaluate the different treatments (i.e. study drug, device, procedure) during the study? If one proves to be clearly more effective than another (or others) during the course of a study, will the study be terminated before the projected total participant population has been enrolled? When will the study end if no important differences are detected?

If no significant obstacles are encountered, the final assessment will take place between January and June 2017. The final analysis will be concluded by December of that same year, while the manuscript will be prepared and disseminated in the Spring of 2018.

3. Background

a) Describe past experimental and/or clinical findings leading to the formulation of the study.

Our team partnered with the Ravenswood City School District in the Winter/Spring of 2013 to implement a 3-hour mindfulness training for district staff (n=163 survey respondents) and an 8-week mindfulness program in nine 3rd and 4th grade classrooms (n=212). In the Fall of 2013 we extended the program to one 7th grade classroom (n=30). The thirty minute mindfulness sessions aimed to improve social-emotional functioning through psychoeducation and mindfulness.

Of approximately 200 staff participants, 163 responded to the survey suggesting appropriate audience engagement. Eighty-one percent of those surveyed either agreed or strongly agreed that after the training they can apply mindfulness skills to themselves, students and colleagues. A majority of the participants, 61%, felt they experienced less challenging emotions, and more positive ones. The training appears to have been helpful for 70% of the participants in establishing an increased awareness of their thoughts, emotions, and physical sensations, which is an essential skill of mindful awareness. A majority of the participants (67%) reported being satisfied with the training overall. More than half of the sample (56%) agreed or strongly agreed they would like to receive further training from our program.

Our second implementation, with 7th graders, included several program enhancements
stemming from our pilot results. We also evaluated practice frequency and obtained quality
assurance feedback from students and the classroom teacher. Specifically we evaluated
student satisfaction with their learning, practice, and understanding of mindfulness.
Classroom teacher feedback was collected on program content, effectiveness of
implementation, and partnership using a 5-point item rating scale. With respect to student
feedback, 88.5% reported enjoying learning about mindfulness, 73.1% enjoyed practicing
mindfulness, 84.6% understood mindfulness, and 42.3% reporting being able to practice on
their own.

We examined pre- and post-score changes of 3rd and 4th graders on the Behavior
Assessment Scale for Children, 2nd Ed. (BASC-2). Statistically significant improvement was
found for students starting in the "at risk" or "clinically significant" category, with changes in
internalizing (anxiety, depression, attention) problems being most consistent. Students "at?
risk" or "clinically significant" with regards to personal adjustment benefited most, with
statistically significant improvement in internalizing problems (depression and anxiety),
emotional symptoms, and personal adjustment.

Student attendance was assessed with 100% of the students attending 5 or more sessions and
nearly 45% attending all 8 sessions. Teacher and students reported that the content was
properly aligned to student age, grade-level, and classroom environment. The teacher
reported having a well-defined role in instruction and classroom management. Further she
felt her skills and input were appropriately utilized during curriculum development and
throughout the program. On the teacher questionnaire, the average program "content" score
was 4.75 (out of 5); the "partnership" rating was 5; and the average program "self-
sustainability" score was 4, referring to the ability of the teacher to continue mindfulness in
the classroom after the program ended.

b) Describe any animal experimentation and findings leading to the formulation of the study.

Findings from animal experimentation have not played a significant role in the formulation of
this study.

Title : The Health and Wellness Curriculum Assessment
Approval Period: 04/24/2015 - 09/23/2015

4. Radioisotopes or Radiation Machines

a) List all standard of care procedures using ionizing radiation (radiation dose received by a subject that is considered part of their normal medical care). List all research procedures using ionizing radiation (procedures performed due to participation in this study that is not considered part of their normal medical care). List each potential procedure in the sequence that it would normally occur during the entire study. More Info

Identify Week/Month of study	Name of Exam	Identify if SOC or Research

b) For research radioisotope projects, provide the following radiation-related information:

Identify the radionuclide(s) and chemical form(s).

For the typical subject, provide the total number of times the radioisotope and activity will be administered (mCi) and the route of administration.

If not FDA approved provide dosimetry information and reference the source documents (package insert, MIRD calculation, peer reviewed literature).

c) For research radiation machine projects, provide the following diagnostic procedures:

For well-established radiographic procedures describe the exam.

For the typical subject, identify the total number of times each will be performed on a single research subject.

For each radiographic procedure, provide the setup and technique sufficient to permit research subject dose modeling. The chief technologist can usually provide this information.

For radiographic procedures not well-established, provide FDA status of the machine, and information sufficient to permit research subject dose modeling.

d) For research radiation machine projects, provide the following therapeutic procedures:

For a well-established therapeutic procedure, identify the area treated, dose per fraction and number of fractions. State whether the therapeutic procedure is being performed as a normal part of clinical management for the research participants's medical condition or whether it is being performed because the research participant is participating in this project.

For a therapeutic procedure that is not well-established, provide FDA status of the machine, basis for dosimetry, area treated, dose per fraction and number of fractions.

5. Devices

a) Please list in the table below all Investigational Devices (including Commercial Devices used off-label) to be used on participants.

5. 1 Device Name : Customized MRI Scan Equipment

Describe the device to be used.

Some of the RF imaging coils, imaging software and other devices used to conduct scans at the Lucas Center and CNI (Stanford Center for Cognitive and Neurobiological Imaging) are not approved by the FDA.

Manufacturer :

Risk : **Non-significant**

Y I confirm the above are true.

Rationale for the device being non-significant risk:

The customized equipment does not currently exist in the market, hence our characterization of it as investigational. However, it has been tested for safety by highly trained Lucas personnel.

Sponsor of Project

Indicate who is responsible for submitting safety reports to the FDA:

Y The sponsor is the device manufacturer.

Ordering, Storage and Control

To prevent the device being used by a person other than the investigator, and in someone other than a research participant: Confirm that the device will be handled according to the SHC/LPCH policy for Investigational New Devices or as appropriate. If no, please provide an explanation. :

Y Confirm?

b) Please list in the table below all IDE Exempt Devices (Commercial Device used according to label, Investigational In Vitro Device or Assay, or Consumer Preference/Modifications/Combinations of Approved Devices) to be used on participants.

5. 1 Device Name : SalivaBio Oral Swab

Describe the device to be used.

This interference-free oral swab device will be used to collect dirunal salivary cortisol samples from participants. Each comes individually wrapped to minimize the possibility of environmental contaminants.

Manufacturer Salimetrics

IDE Exemption

Y **This is a legally marketed device being used in accordance with its labeling.**

5. 2 Device Name : Safiro Ambulatory PSG Monitoring Syst

Describe the device to be used.

**This ambulatory device records EEG, EMG, expiratory/inspiratory nasal airway pressure, nasal/oral airflow, finger pulse oximetry,electrocardiogram ECG, movements of the rib cage and abdomen, snoring, body position and bilateral anterior tibialis
EMG.**

Manufacturer Compumedics

IDE Exemption

Y **This is a legally marketed device being used in accordance with its labeling.**

6. Drugs, Reagents, or Chemicals and Devices

a) Please list in the table below all investigational drugs, reagents or chemicals to be administered to participants.

b) Please list in the table below all commercial drugs, reagents or chemicals to be administered to

participants.

7. Medical Equipment for Human Subjects and Laboratory Animals

If medical equipment used for human patients/participants is also used on animals, describe such equipment and disinfection procedures.

The bed/table and accessories that are used for the animals is different than the table humans use. Physiologic monitoring equipment is cleaned with a commercial disinfectant such as Roccal, Conflick, Sani-Wipes, or a 10% Bleach solution. All RF coils and positioning accessories are wrapped in plastic wrap or plastic bags for use with animals. Everything, even if it is animal use only, is cleaned with the above disinfectants after every use even if they are wrapped in plastic. The Lucas Center is checked yearly by several groups at Stanford who approve animal research in human systems: Stanford Health & Safety. We are reviewed by: Stanford APLAC panel; USDA; NIH; and Aaalac.

8. Participant Population

a) **State the following:** (**i**) **the number of participants expected to be enrolled at Stanford-affiliated site**(s); (**ii**)**the total number of participants expected to enroll at all sites;** (**iii**) **the type of participants** (**i.e. students, patients with certain cancer, patients with certain cardiac condition**) **and the reasons for using such participants.**

i. Approximately 900 (Roughly 800 Ravenswood 3rd and 5th graders + roughly 100 controls (also 3rd and 5th graders) from a neighboring school district, Orchard School District, in San Jose, CA.

ii. Approximately 900

iii. See below:

The entire Ravenswood City School district will be receiving the Health and Wellness Curriculum as part of their regular instruction, regardless of our research. Of the 3500 students enrolled in Ravenswood, 800 3rd and 5th graders will be recruited for our study. This 800 student sample will receive academic and neurobehavioral assessments.

From those 800 students, a 100 student sub-sample will be recruited for cognitive, sleep, and endocrinology assessments. From this 100 student sub sample, at least 30 students will be recruited to participate in the neuroimagining component of the study.

A socioeconomically matched control group of 100 students will be recruited from neighboring school district that are not receiving the Health and Wellness Curriculum. These 100 students will complete all assessments (academic, neurobehavioral, cognitive, sleep, endocrinology, and neuroimaging).

Both experimental and comparison groups must demonstrate equivalence except for the curriculum. Ideally, a randomization procedure would assign some children to the curriculum and others to the comparison group. In a community, naturalistic study, such as this one, this is not feasible because we predict that changes in the school environment would impact all children favorably.

b) **State the age range, gender, and ethnic background of the participant population being recruited.**

Age: 7-13

Gender: Even distribution anticipated.

Ethnic Background:
(According to the Ravenswood City School District's Website)

78% Hispanic
10% African American
10% Pacific Islander
02% Other

c) State the number and rationale for involvement of potentially vulnerable subjects in the study (including children, pregnant women, economically and educationally disadvantaged, decisionally impaired, homeless people, employees and students). Specify the measures being taken to minimize the risks and the chance of harm to the potentially vulnerable subjects and the additional safeguards that have been included in the protocol to protect their rights and welfare.

Because the study examines an in-school curriculum, there are no alternatives to grade school aged students in their classrooms. Participants will be given contact information for the study PI's and will know how to get in touch will study staff. IF further intervention is needed, participants will be provided with appropriate referrals to community agencies or treatment facilities.

Our target population includes recent immigrants who may be at risk for deportation. We ask that the IRB wave the payment requirement to collect social security numbers from parents of child subjects.

d) If women, minorities, "https://stanfordmedicine.box.com/shared/static/4aj1fth309551do70wg6t06pukl98as6.pdf" target="_blank"non-English speaking individuals, or children are not included, a clear compelling rationale must be provided (e.g., disease does not occur in children, drug or device would interfere with normal growth and development, etc.).

N/A

e) State the number, if any, of participants who are laboratory personnel, employees, and/or students. They should render the same written informed consent. If payment is allowed, they should also receive it. Please see Stanford University policy.

N/A

f) State the number, if any, of participants who are healthy volunteers. Provide rationale for the inclusion of healthy volunteers in this study. Specify any risks to which participants may possibly be exposed. Specify the measures being taken to minimize the risks and the chance of harm to the volunteers and the additional safeguards that have been included in the protocol to protect their rights and welfare.

All participants in this study may be considered healthy volunteers, as they have not been recruited based on any known medical conditions, and will receive the curriculum independently of the research.

g) Describe your plan to identify and recruit potential participants including who will inform them about the study and how they will be initially contacted by the researchers (e.g., https://med.stanford.edu/spectrum/researcher-resources/participant-engagement.html Research Engagement services; chart review; treating physician; ads including social media posts). All final or revised recruitment materials must be approved by the IRB before use. Contacting potential participants is not permitted prior to IRB approval. See https://stanfordmedicine.box.com/shared/static/8uebsdjrrqjyauanjp9i9d0gm1i480co.pdf Recruitment Guidance for additional information.

Approximately 800 3rd and 5th grade students in the Ravenswood City School District have been identified for recruitment, based solely on their enrollment in a Ravenswood City school. For the 100 student control group (also 3rd and 5th graders) from Orchard School District in San Jose. The health and wellness curriculum is not provided in the Orchard School District.

h) Inclusion and Exclusion Criteria.

Identify inclusion criteria.

All 3rd and 5th grade students enrolled at the Ravenswood City School District at the first time point are by eligible to participate.

Identify exclusion criteria.

For typically-developing participants, exclusion criteria for the fMRI component of our study are as follows:

1. History of trauma involving head injury.
2. Metal in or on the body that cannot be removed(braces, piercings or tattoos).
3. Consistent exposure to metal(i.e. hobbies grinding metal).
4. Pregnancy.
5. Left-handedness.

Students who demonstrate the inability to follow all study procedures during a specific assessment will be excluded from that portion of the study.

i) Describe your screening procedures, including how qualifying laboratory values will be obtained. If you are collecting personal health information prior to enrollment (e.g., telephone screening), please request a waiver of authorization for recruitment (in section 15).

Screening will only occur for the neuroimaging portion of the study. Eligibility will be established through the Medical and Developmental History form, MRI screening form, and handedness form.

j) Describe how you will be cognizant of other protocols in which participants might be enrolled. Please explain if participants will be enrolled in more than one study.

On the student demographics form (attached in section 16), participants are asked about whether or not they are participating in other research. This possibility is unlikely, as there are no known protocols actively recruiting the population outside of this study.

k) Payment/reimbursement. Explain the amount and schedule of payment or reimbursement, if any, that will be paid for participation in the study. Substantiate that proposed payments are reasonable and commensurate with the expected contributions of participants and that they do not constitute undue pressure on participants to volunteer for the research study. Include provisions for prorating payment. See payment considerations

Upon completion of each assessment at the first, second and third time points, students will receive the following payments depending on which combination of assessments they complete:

$25 dollars each time for completing the academic and neurobehavioral questionnaires ($75 total).

$50 each time for completing the academic, neurobehavioral AND cognitive assessments ($150 total).

$75 dollars each time for completing the academic, neurobehavioral, and cognitive assessments AND saliva collection ($225 total).

$150 each time for completing the academic, neurobehavioral, and cognitive assessments AND the saliva collection AND the sleep assessment ($450 total).

$250 each time for completing all three questionnaires AND the saliva collection AND the sleep assessment AND the brain scans ($750 total).

For the Bart task, participants will receive monetary reward for their grand total earnings from the task, subject to a maximum of $15.

Participants will also complete a Delay Discounting Task outside of the MRI scanner. During each trial of the task, participants will be presented with a choice between an immediate monetary reward ($11-80), and a larger reward ($25-85) delayed by 7-186 days. Participants will receive 10% of the monetary reward from a randomly selected trial. Participants will be paid at the end of the session if the chosen trial was an immediate reward. For delayed rewards, participants will be mailed payment at the delay specified in the

trial.

Control participants from Orchard School District:
$25 each time he/she completes the social/emotional/academic assessment ($75 total).

$25 each time he/she completes the intellectual function assessment ($75 total).

$25 each time he/she completes the saliva collection ($75 total)

$75 each time he/she completes the sleep assessment ($225 total)

$100 each time he/she completes the brain scan ($300 total)

l) Costs. Please explain any costs that will be charged to the participant.

There is no cost for participation outside of minimal travel expenses for those students participating in the neuroimaging component of the study, for which they can be reimbursed.

m) Estimate the probable duration of the entire study. Also estimate the total time per participant for: (i) screening of participant; (ii) active participation in study; (iii) analysis of participant data.

The probable duration of the entire study is 4 years.

For the academic and neurobehavioral assessments, active participation time will be approximately 2 hours at three different points in time, for a total commitment of about 6 hours.

For the cognitive assessments, active participation time will be about an hour at three different points in time, for a total commitment of about three hours.

For the salivary cortisol sampling, active participation time will be five short collections over the course of two consecutive days at three points in time, for a total of roughly one week of active participation.

For the sleep assessments, training will amount to no more than three hours over the course of (up to) several days at the family's convenience, and assessment will require one night of monitoring at three points in time. Total estimate is about one week.

For the neuroimaging component, participants will schedule a two to three hour session in the Lucas Center at three time points. Total time commitment is estimated at one full day.

9. Risks

a) For the following categories include a scientific estimate of the frequency, severity, and reversibility of potential risks. Wherever possible, include statistical incidence of complications and the mortality rate of proposed procedures. Where there has been insufficient time to accumulate significant data on risk, a statement to this effect should be included. (In describing these risks in the consent form to the participant it is helpful to use comparisons which are meaningful to persons unfamiliar with medical terminology.)

The risks of the Investigational devices.

For MRI scans, there is a minimal non-significant risk associated with use of the custom-developed imaging coils, imaging software and other non-FDA approved devices used at the Lucas Center.

Magnetic fields do not cause harmful effects at the levels used in the MRI machine. However, the MR scanner uses a very strong magnet that will attract some metals and affect some electronic devices. If the child has a cardiac pacemaker or any other biomedical device in or on his or her body, it is very important that he or she tell the operator/investigator immediately. As metallic objects may experience a strong

attraction to the magnet, it is also very important that parents notify the operator of any metal objects (especially surgical clips), devices, or implants that are in or on the child's body before entering the magnet room. All such objects must be removed (if possible) before entering the magnet room. In some cases, having those devices means thw child should not have an MRI scan performed. In addition, watches and credit cards should also be removed as these could be damaged. The child will be provided a way to secure these items. If the child has any history of head or eye injury involving metal fragments, if he or she has ever worked in a metal shop, or could be pregnant, parents should notify the operator/investigator.

The sleep equipment may be uncomfortable but the desensitization process is in place to minimize any discomfort. There is a minimal risk of skin irritation as a result of the gel applied to adhere the cap. Should this occur we will immediately stop the procedure.

The risks of the Investigational drugs. Information about risks can often be found in the Investigator's brochure.

N/A

The risks of the Commercially available drugs, reagents or chemicals. Information about risks can often be found in the package insert.

N/A

The risks of the Procedures to be performed. Include all investigational, non-investigational and non-invasive procedures (e.g., surgery, blood draws, treadmill tests).

N/A

The risks of the Radioisotopes/radiation-producing machines (e.g., X-rays, CT scans, fluoroscopy) and associated risks.

N/A

The risks of the Physical well-being.

The subjects will be practicing mindful stretching, which is a form of exercise. With any physical exercise there is a minimal risk of harm. The postures included in the study are reported to be safe for the youth included in this study based upon existing mindfulness literature.

The risks of the Psychological well-being.

Subjects may experience minimal psychological discomfort as a result of practicing meditation initially. Over time, on average, subjects engaged in these practices have positive outcomes

There are no significant risks to the participant from the interviewing, testing, or psychophysiological measures. They may have some anxiety or discomfort in answering the questions or doing the tests. Other risks relate to finding out that they may have a genetic or medical abnormality that they had not been aware of before. This knowledge could cause psychological stress to them or their family and possibly affect their health insurance coverage in the future.

The risks of the Economic well-being.

N/A

The risks of the Social well-being.

Subjects who may be adverse to group settings may experience minimal discomfort; however, the curriculum may facilitate a positive experience for subjects, lessening any initial discomfort they may experience.

Overall evaluation of Risk.

Low - innocuous procedures such as phlebotomy, urine or stool collection, no therapeutic agent, or safe therapeutic agent such as the use of an FDA approved drug or device.

b) If you are conducting international research, describe the qualifications/preparations that enable you to both estimate and minimize risks to participants. Provide an explanation as to why the research must be completed at this location and complete the [LINKFORINTERNATIONALREASEARCHFORM] International Research Form. If not applicable,

enter N/A.

N/A

c) **Describe the planned procedures for protecting against and minimizing all potential risks. Include the means for monitoring to detect hazards to the participant (and/or to a potential fetus if applicable). Include steps to minimize risks to the confidentiality of identifiable information.**

Ongoing supervision and research team meetings will be conducted to consult regarding any potential risks. All subject data will be stored in locked filing cabinets and password secured computers in a locked research lab on Stanford University's campus.

Children not participating in the study will be provided an alternative activity by the school during the time of assessments which will amount to approximately 4 class periods for each time point. The assessments will not interfere with regular classroom time that would otherwise be devoted to academic subjects, and will only be administered during designated health and wellness periods or elective classes (based on consultation with school principals).

Research staff are well trained to use protocols and procedures to ensure that the MRI equipment is used correctly to minimize any risks. The imaging coils and software are also tested for safety. Subjects are instructed that they can alert the operator that they need help or need to ask a question by squeezing a hand-held device during the MRI scan. Scans will be terminated at a subject's or parent's request or if the console operator or research staff in the scanner detect significant subject discomfort. Subjects and their parents will be asked to notify staff immediately if they encounter any problems. If a serious adverse event occurs during the study, research staff will advise the subject's parents to discontinue participation and a study physician will confer with the subject's personal physician if the parent so chooses.

Research staff are well trained to use protocols and procedures to ensure that the ambulatory PSG equipment is used correctly as well. Families will be trained at their own pace on how to conduct this assessment, and will not be instructed to do so until they feel comfortable. Subjects are again advised to terminate the procedure by removing the cap and electrodes should they feel any discomfort while the sleep assessment is being conducted. A significant part of our preparation for the PSG involves working with participants and their families using systematic desensitization in order to make them more comfortable with the PSG process.

d) **Explain the point at which the experiment will terminate. If appropriate, include the standards for the termination of the participation of the individual participant Also discuss plans for ensuring necessary medical or professional intervention in the event of adverse effects to the participants.**

At the discretion of the protocol director, subjects may be taken out of this study due to unanticipated circumstances. Some possible reasons for withdrawing a subject from the study include: The subject fails to follow instructions or perform specified tasks; The investigator decides that continuation could be harmful to the subject; The subject requires treatment not allowed in the study; The study is cancelled; Other administrative reasons.

If anything from the brain scans, cortisol sampling, physiological assessments, interviewing, or testing is learned we will share that information with the family. Additionally, if anything happens during the interviewing, testing, or brain scan, we will report it to the IRB, and refer the patient to the appropriate care.

e) **Data Safety and Monitoring Plan (DSMP). See guidance on Data Safety and Monitoring.**

A Data and Safety Monitoring Plan (DSMP) is required for studies that present Medium or High risk to participants. (See Overall Evaluation of Risk above). If Low Risk, a DSMP may not be necessary. Multi-site Phase III clinical trials funded by NIH require the DSM Plan to have a Data Safety Monitoring Board or Committee (DSMC or DSMB). The FDA recommends that all multi-site clinical trials that involve interventions that have potential for greater than minimal risk to study participants also have a DSMB or DSMC.

The role of the DSMC or DSMB is to ensure the safety of participants by analyzing pooled data from all sites, and to oversee the validity and integrity of the data. Depending on the degree of risk and the complexity of the protocol, monitoring may be performed by an independent committee, a board (DSMC/DSMB), a sponsor's Data Safety Committee (DSC), a Medical Monitor, a sponsor's safety officer, or by the Protocol Director (PD).

Describe the following:

What type of data and/or events will be reviewed under the monitoring plan, e.g. adverse events, protocol deviations, aggregate data?

This is a low-risk study, and therefor does not have a monitoring plan.

Identify who will be responsible for Data and Safety Monitoring for this study, e.g. Stanford Cancer Institute DSMC, an independent monitoring committee, the sponsor, Stanford investigators independent of the study, the PD, or other person(s). a style='font-style:italic;color:#0000CC' href= https://rco.sites.stanford.edu/eprotocol/ephelp_dsmp#dsmp2 more...

Protocol Director Victor Carrion, MD, will be responsible for appropriate characterization of unanticipated risks to participants or others and adverse events. He has decades of working with sensitive patient populations like those involved in this study. Study personnel will record these events, logging them in official records so that they are kept with the experiment data. Adverse events will also be reported to the IRB, and if necessary, participants will be referred to appropriate care.

Provide the scope and composition of the monitoring board, committee, or safety monitor, e.g., information about each member's relevant experience or area of expertise. If the Monitor is the Stanford Cancer Center DSMC or the PD, enter N/A.

This is a low-risk study, and therefor does not have a monitoring board or committee.

Confirm that you will report Serious Adverse Events (SAEs), Suspected Unexpected Serious Adverse Reactions (SUSARs), or Unanticipated Problems (UPs) to the person or committee monitoring the study in accordance with Sponsor requirements and FDA regulations.

Any Serious Adverse Events (SAEs), Suspected Unexpected Serious Adverse Reactions (SUSARs), or Unanticipated Problems (UPs) will be reported to the Protocol Director, Victor Carrion, MD.

If applicable, how frequently will the Monitoring Committee meet? Will the Monitoring Committee provide written recommendations about continuing the study to the Sponsor and IRB?

This is a low-risk study, and therefor does not have a monitoring committee.

Specify triggers or stopping rules that will dictate when the study will end, or when some action is required. If you specified this in Section 2g [Study Endpoints], earlier in this application enter 'See 2g'.

See 2g

Indicate to whom the data and safety monitoring person, board, or committee will disseminate the outcome of the review(s), e.g., to the IRB, the study sponsor, the investigator, or other officials, as appropriate.

The protocol Director, Victor Carrion, will disseminate the outcome of the reviews to the IRB as needed.

Select One:

Y The Protocol Director will be the only monitoring entity for this study.
This protocol will utilize a board, committee, or safety monitor as identified in question #2 above.

f) Special Participant Populations

Children's Findings FDA. As your research includes children and an investigational drug/device or a

Title : The Health and Wellness Curriculum Assessment
Approval Period: 04/24/2015 - 09/23/2015

commercial device is being studied, please select one or more regulatory categories (50.51 through 50.54) below that your research falls under and provide the necessary rationale for each determination. See full regulation citation.

Y 50.51 Clinical Investigations not involving greater than minimal risk. The research must present no greater than minimal risk to children and adequate provisions must be made for soliciting the assent of the children and the permission of their parents or guardians. Please provide rationale for the above statement.

Rationale for category selected above

For MRI scans, there is a minimal non-significant risk associated with use of the custom-developed imaging coils, imaging software and other non-FDA approved devices used at the Lucas Center. Magnetic fields do not cause harmful effects at the levels used in the MRI machine. There is no literature to support the assumption of any significant risk in use of the diurnal salivary cortisol collection swabs. The sleep equipment may be uncomfortable but the desensitization process is in place to minimize any discomfort. There is a minimal risk of skin irritation as a result of the gel applied to adhere the cap. Should this occur we will immediately stop the procedure.

10. Benefits

a) **Describe the potential benefit(s) to be gained by the participants or by the acquisition of important knowledge which may benefit future participants, etc.**

Subjects may benefit indirectly through the information learned in their cognitive and behavioral evaluation.

11. Privacy and Confidentiality

Privacy Protections

a) **Describe the setting and method (e.g. crowded waiting room, patient exam room, telephone or email communication) in which interactions will occur and how the privacy interests of participants will be maintained. Note, high risk data such as PHI must be sent via "Secure:" email per https://uit.stanford.edu/security/hipaa/email-policy Stanford policy.**

All HIPAA regulations will be followed. Arrangements for data collection will be made via telephone. Subjects participating in the cognitive, cortisol, and sleep portions of the study will be assessed in the privacy of their own homes or in the Lucas Center for Imaging at Stanford.

Confidentiality Protections

b) **Specify PHI (Protected Health Information). PHI is health information linked to HIPAA identifiers (see above). List BOTH health information AND HIPAA identifiers. If you are using STARR, use the Data Privacy Attestation to ensure that your request will match your IRB-approved protocol. Be consistent with information entered in section 15a.**

PHI such as name, age, contact information and medical and demographic history will be obtained and deidentified using unique study IDs. Salivary cortisol samples will be obtained and deidentified using these same study IDs. This information will not be

Title : The Health and Wellness Curriculum Assessment
Approval Period: 04/24/2015 - 09/23/2015

disclosed to others.

Please see the attached Medical and Demographic History form for a complete list
of the PHI
being collected.

c) **You are required to comply with University Policy that states that ALL electronic devices: computers (laptops and desktops; OFFICE or HOME); smart phones; tablets; external hard disks, USB drives, etc. that may hold identifiable participant data will be password protected, backed up, and encrypted.**

Stanford University IT approved platforms (https://uit.stanford.edu/guide/riskclassifications https://uit.stanford.edu/guide/riskclassifications) should be used for data management. Consult with your Department IT representative for more information.For data security policies and links to encrypt your devices see http://med.stanford.edu/irt/security and target=_blankhttp://www.stanford.edu/group/security/securecomputing/mobile_devices.html. Additionally, any PHI data on paper must be secured in a locked environment.

By checking this box, You affirm the aforementioned. Y

Data will be labeled with each subject name linked to a unique numeric subject id.
There will
be a password protected computer file containing the master list. The de-
identification will be
performed by members of the research team.

d) **Describe how data or specimens will be labeled (e.g. name, medical record number, study number, linked coding system) or de-identified. If you are de-identifying data or specimens, who will be responsible for the de-identification? If x-rays or other digital images are used, explain how and by whom the images will be de-identified.**

For each subject, a folder will be created that contains all interviews of the subject
and family,
cognitive tests, behavioral ratings and checklists, and imaging data. Subject
identity
will be numerically coded on the subject folders and will be stored in a locked file
cabinet
accessible only to study personnel. Data will be entered into the computerized
database using
the subject ID number.

e) **Indicate who will have access to the data or specimens (e.g., research team, sponsors, consultants) and describe levels of access control (e.g., restricted access for certain persons or groups, access to linked data or specimens).**

Lab technicians at Salimetrics will have access to deidentified salivary cortisol
samples labeled
by unique study IDs. The Salimetrics lab will not have access to any PHI
associated with these
samples. The samples will be destroyed after analysis. Results of this analysis will
be reported
to our research team, after which Salimetrics will not maintain their own records.

The Salimetrics lab is CLIA certified. The Stanford research team does not have a
Business
Associate Agreement with Salimetrics. In this capacity, Salimetrics is acting as a
vendor,
providing standard, confidential services as per their usual business model.

f) **If data or specimens will be coded, describe the method in which they will be coded so that study participants' identities cannot be readily ascertained from the code.**

Data and specimens will be coded by the order in which they were collected by our research team.

g) If data or specimens will be coded, indicate who will maintain the key to the code and describe how it will be protected against unauthorized access.

Only core research staff will have access to the key which will be locked within the ELSPAP Lab.

h) If sharing data with others, describe how data will be transferred or transmitted (**e.g., file transfer software, file sharing, email**). **If transmitted via electronic networks, confirm a Stanford University IT approved platform will be used** (**see https://uit.stanford.edu/guide/riskclassifications https://uit.stanford.edu/guide/riskclassifications**) **or that data will be encrypted while in transit. Additionally, confirm appropriate agreements are in place to allow for the sharing** (**see https://ico.stanford.edu/stanford-researchers/who-will-handle-my-agreement https://ico.stanford.edu/stanford-researchers/who-will-handle-my-agreement**). **If using or sharing PHI, refer to the following policies: https://uit.stanford.edu/security/hipaa https://uit.stanford.edu/security/hipaa.**

Data will be transferred through secure FTP transfer method or the Stanford Medicine Box. In case we use email, we will password protect documents.

i) How will you educate research staff to ensure they take appropriate measures to protect the privacy of participants and the confidentiality of data or specimens collected (**e.g. conscious of oral and written communications, conducting insurance billing, and maintaining paper and electronic data**)**?**

All staff are CITI and HIPAA trained. We will also hold regular meetings to go over confidentiality issues.

12. Potential Conflict of Interest

Investigators are required to disclose any outside interests that reasonably appear to be related/li to this protocol.

Outside Interest Tasks

Investigators	Role	Potential COI?	Date Outside Interest Answered	Date OPACS Disclosure Submitted	COI Review Determination
Victor G. Carrion	PD	N	08/20/2014	N/A	N/A
Vinod Menon	COPD	N	08/25/2014	N/A	N/A
Ruth O'Hara	OC	N	08/21/2014	N/A	N/A

Title : The Health and Wellness Curriculum Assessment
Approval Period: 04/24/2015 - 09/23/2015

13. Consent Background

13. 1 Consent HWC Parent Consent (Revised 10.14.14)

Check if VA related

a) Describe the informed consent process. Include the following.

- **i) Who is obtaining consent? (The person obtaining consent must be knowledgeable about the study.)**
- **ii) When and where will consent be obtained?**
- **iii) How much time will be devoted to consent discussion?**
- **iv) Will these periods provide sufficient opportunity for the participant to consider whether or not to participate and sign the written consent?**
- **v) What steps are you taking to minimize the possibility of coercion and undue influence?**
- **vi) If consent relates to children and if you have a reason for only one parent signing, provide that rationale for IRB consideration.**

i. Consent will obtained by trained members of the research staff. ii. Consent will be obtained from parents at one of several information sessions held at their children's schools. iii. As much time as is needed to answer all parents and caregivers' questions. iv. Yes. v. Parents will have multiple opportunities to consent, and will have the ability to review information on their own time before making a final decision. vi. N/A

b) What is the Procedure to assess understanding of the information contained in the consent? How will the information be provided to participants if they do not understand English or if they have a hearing impairment? See HRPP Chapter12.2 for guidance.

The employees and students at the Ravenswood City School District are English speakers. The Protocol Director, Victor Carrion, is a fluent Spanish speaker, and will be conducting information sessions with families personally. Research staff interfacing with Spanish speaking families will also be fluent Spanish speakers. A native Spanish speaker on the research team, Veronica Alvarez will be providing verbatim translations of consent forms for non-English speakers. In the event that there is another language barrier or hearing impaired participant, appropriate arrangements will be made to bring a translator or interpreter to assist in the consent process.

c) What steps are you taking to determine that potential participants have the capacity to participate in the decision-making process? If your study may enroll adults who are unable to consent, describe (i) how you will assess the capacity to consent, (ii) what provisions will be taken if the participant regains the capacity to consent,(iii) who will be used as a legally authorized representative, and (iv) what provisions will be made for the assent of the participant.

Consent is being obtained from both parents as well as child participants. In the event that a child does not live with his or her biological parents, consent will be obtained from adoptive parents, foster parents, or legal guardians. All families will complete a comprehensive medical and demographic history form (attached in section 16) that will identify any confounds that might invalidate consent.

13. 2 Consent HWC Parent Consent (Spanish)

Check if VA related

a) Describe the informed consent process. Include the following.

- **i) Who is obtaining consent? (The person obtaining consent must be knowledgeable about the study.)**
- **ii) When and where will consent be obtained?**
- **iii) How much time will be devoted to consent discussion?**
- **iv) Will these periods provide sufficient opportunity for the participant to consider whether or not to participate and sign the written consent?**
- **v) What steps are you taking to minimize the possibility of coercion and undue influence?**
- **vi) If consent relates to children and if you have a reason for only one parent signing, provide that rationale for IRB consideration.**

i. Consent will obtained by trained members of the research staff. ii. Consent will be obtained from parents at one of several information sessions held at their children's schools. iii. As much time as is needed to answer all parents and caregivers' questions. iv. Yes. v. Parents will have multiple opportunities to consent, and will have the ability to review information on their own time before making a final decision. vi. N/A

b) What is the Procedure to assess understanding of the information contained in the consent? How will the information be provided to participants if they do not understand English or if they have a hearing impairment? See HRPP Chapter12.2 for guidance.

Title : The Health and Wellness Curriculum Assessment
Approval Period: 04/24/2015 - 09/23/2015

The employees and students at the Ravenswood City School District are English speakers. The Protocol Director, Victor Carrion, is a fluent Spanish speaker, and will be conducting information sessions with families personally. Research staff interfacing with Spanish speaking families will also be fluent Spanish speakers. A native Spanish speaker on the research team, Veronica Alvarez will be providing verbatim translations of consent forms for non-English speakers. In the event that there is another language barrier or hearing impaired participant, appropriate arrangements will be made to bring a translator or interpreter to assist in the consent process.

c) **What steps are you taking to determine that potential participants have the capacity to participate in the decision-making process? If your study may enroll adults who are unable to consent, describe (i) how you will assess the capacity to consent, (ii) what provisions will be taken if the participant regains the capacity to consent,(iii) who will be used as a legally authorized representative, and (iv) what provisions will be made for the assent of the participant.**

Consent is being obtained from both parents as well as child participants. In the event that a child does not live with his or her biological parents, consent will be obtained from adoptive parents, foster parents, or legal guardians. All families will complete a comprehensive medical and demographic history form (attached in section 16) that will identify any confounds that might invalidate consent.

13. 3 Consent — Questionnaire Consent- Final Version

Check if VA related

a) **Describe the informed consent process. Include the following.**
- i) **Who is obtaining consent? (The person obtaining consent must be knowledgeable about the study.)**
- ii) **When and where will consent be obtained?**
- iii) **How much time will be devoted to consent discussion?**
- iv) **Will these periods provide sufficient opportunity for the participant to consider whether or not to participate and sign the written consent?**
- v) **What steps are you taking to minimize the possibility of coercion and undue influence?**
- vi) **If consent relates to children and if you have a reason for only one parent signing, provide that rationale for IRB consideration.**

Research study staff trained in human subjects research at Stanford will obtain consent. Consent will be obtained in meetings with parents or by sending the consent home to parents. Research staff will stress that participation is voluntary and subjects can withdraw at any time without penalty. IRB has already approved one parent signing the consent (based on the study population where it is often not possible to reach both parents).

b) **What is the Procedure to assess understanding of the information contained in the consent? How will the information be provided to participants if they do not understand English or if they have a hearing impairment? See HRPP Chapter12.2 for guidance.**

Research team will provide cover letter that summarizes consent in very plain language and provides contact number for questions. Spanish speakers will be provided a written Spanish translation of the consent form (see Spanish version of the consent).

c) **What steps are you taking to determine that potential participants have the capacity to participate in the decision-making process? If your study may enroll adults who are unable to consent, describe (i) how you will assess the capacity to consent, (ii) what provisions will be taken if the participant regains the capacity to consent,(iii) who will be used as a legally authorized representative, and (iv) what provisions will be made for the assent of the participant.**

The child's parent or legal guardian will be the legally authorized representative. An assent form will be provided for the child participant and discussed with the child at the time of collecting data. Consenting parent and child will be allowed to ask questions and confirm understanding.

13. 4 Consent — Questionnaire Consent (Spanish)-Final Version

Check if VA related

a) **Describe the informed consent process. Include the following.**
- i) **Who is obtaining consent? (The person obtaining consent must be knowledgeable about the study.)**
- ii) **When and where will consent be obtained?**
- iii) **How much time will be devoted to consent discussion?**
- iv) **Will these periods provide sufficient opportunity for the participant to consider whether or not to participate and sign the written consent?**
- v) **What steps are you taking to minimize the possibility of coercion and undue influence?**

Title : The Health and Wellness Curriculum Assessment
Approval Period: 04/24/2015 - 09/23/2015

vi) If consent relates to children and if you have a reason for only one parent signing, provide that rationale for IRB consideration.

Prior to participation in the study, research study staff will obtain parental consent at the school and/or at child's home and/or Stanford campus for the questionnaire component of this study. A cover letter will also be sent home describing the study. Study staff will emphasize that participation is entirely voluntary and they can withdraw at any time without penalty.

b) What is the Procedure to assess understanding of the information contained in the consent? How will the information be provided to participants if they do not understand English or if they have a hearing impairment? See HRPP Chapter12.2 for guidance.

Contact information for Research study staff will be provided. Staff will be available to discuss the questionnaire components with the parent and answer any questions they have. Spanish translations of the consent form will be provided to parents if needed and bi-lingual Spanish speakers from the research staff will be available to speak with parents.

c) What steps are you taking to determine that potential participants have the capacity to participate in the decision-making process? If your study may enroll adults who are unable to consent, describe (i) how you will assess the capacity to consent, (ii) what provisions will be taken if the participant regains the capacity to consent,(iii) who will be used as a legally authorized representative, and (iv) what provisions will be made for the assent of the participant.

This component of the study only involves questionnaires. The language contained in the consent form has been simplified for parents from low SES backgrounds. IRB has previously determined that consent must be obtained from only one parent based on the study population where it is often difficult or impossible to locate both parents for consent.

13. 5 Waiver of Documentation English Questionnaire Consent_Waiver &Alteration

Check if VA related

a) Describe the informed consent process. Include the following.

i) Who is obtaining consent? (The person obtaining consent must be knowledgeable about the study.)

ii) When and where will consent be obtained?

iii) How much time will be devoted to consent discussion?

iv) Will these periods provide sufficient opportunity for the participant to consider whether or not to participate and sign the written consent?

v) What steps are you taking to minimize the possibility of coercion and undue influence?

vi) If consent relates to children and if you have a reason for only one parent signing, provide that rationale for IRB consideration.

Informed Consent forms (with a waiver of documentation of consent and an alteration of HIPAA authorization) will be sent home with students for their parents detailing the in-school questionnaire part of the study. If parents have additional questions or would like to opt their children out of the study, they may contact school staff or research staff to provide written opt out. The informed consent forms will emphasize that participation is voluntary and will not impact their child's education or access to services. We are requesting the Waiver and Alteration of HIPAA because most of the parents in our intended sample are extremely difficult to reach and secure signatures.

b) What is the Procedure to assess understanding of the information contained in the consent? How will the information be provided to participants if they do not understand English or if they have a hearing impairment? See HRPP Chapter12.2 for guidance.

The informed consent form will provide contact information to parents if they have additional questions about the questionnaire part of the study. The informed consent forms will also be provided in English and Spanish.

c) What steps are you taking to determine that potential participants have the capacity to participate in the decision-making process? If your study may enroll adults who are unable to consent, describe (i) how you will assess the capacity to consent, (ii) what provisions will be taken if the participant regains the capacity to consent,(iii) who will be used as a legally authorized representative, and (iv) what provisions will be made for the assent of the participant.

Parents and legally authorized representatives will be allowed to make the determination of participation in this study. Parents will have the opportunity to ask additional questions about the study if they wish. We will also discuss the study with the student participants and determine their understanding, and we will obtain written Assent after they have the opportunity to ask questions.

Select ALL applicable regulatory criteria for a Waiver of Documentation and provide a protocol-specific justification:

1) **45 CFR 46.117(c)(1)(i)., that the only record linking the participants and the research would be the consent document, and the principal risk would be potential harm resulting from a breach of confidentiality; each participant (or legally authorized representative) will be asked whether he/she wants documentation linking the participant with the research, and the participant's wishes govern.**

2) **45 CFR 46.117(c)(1)(ii)., that the research presents no more than minimal risk of harm to participants and involves no procedures for which written consent is normally required outside of the research context.**

3) **45 CFR 46.117(c)(1)(iii)., if participants or legally authorized representatives (LAR) are members of a distinct cultural group in which signing forms is not the norm, the research presents no more than minimal risk and there is an appropriate alternative mechanism for documenting that informed consent was obtained.**

4) Y **21 CFR 56.109(c)(1)., presents no more than minimal risk of harm to participants and involves no procedures for which written consent is normally required outside of the research context.**

Rationale for above selection:

For this portion of our study, which only involves answering some questionnaires about thoughts, feelings and academics, we request a Waiver of Documentation of Consent and Alteration of HIPPA Authorization because it is extremely difficult to secure a signature from parents in this population as many work more than one job, family life can be very unpredictable and many parents are not actively involved in school activities. The questionnaires present no more than minimal risk of harm. If a parent wishes to have his or her child opt out of the study, they may contact their principal or research study staff to notify them. Research study staff will follow up with as many parents as possible to complete the additional questionnaires (like Medical and Developmental History Form) covered through informed consent without signature. The original Questionnaire Consent will still be used to secure signatures from parents when possible, because we will need signed consent to access their child's academic records and to comply with FERPA.

13. 6 Waiver of Documentation Spanish Questionnaire Consent Waiver &Alteration

Check if VA related

a) **Describe the informed consent process. Include the following.**

i) **Who is obtaining consent? (The person obtaining consent must be knowledgeable about the study.)**

ii) **When and where will consent be obtained?**

iii) **How much time will be devoted to consent discussion?**

iv) **Will these periods provide sufficient opportunity for the participant to consider whether or not to participate and sign the written consent?**

v) **What steps are you taking to minimize the possibility of coercion and undue influence?**

vi) **If consent relates to children and if you have a reason for only one parent signing, provide that rationale for IRB consideration.**

Informed Consent forms (with a waiver of documentation of consent and an alteration of HIPAA authorization) will be sent home with students for their parents detailing the in-school questionnaire part of the study. If parents have additional questions or would like to opt their children out of the study, they may contact school staff or research staff to provide written opt out. The informed consent forms will emphasize that participation is voluntary and will not impact their child's education or access to services. We are requesting the Waiver and Alteration of HIPAA because most of the parents in our intended sample are extremely difficult to reach and secure signatures.

b) **What is the Procedure to assess understanding of the information contained in the consent? How will the information be provided to participants if they do not understand English or if they have a hearing impairment? See HRPP Chapter12.2 for guidance.**

The informed consent form will provide contact information to parents if they have additional questions about the questionnaire part of the study. The informed consent forms will also be provided in English and Spanish.

c) **What steps are you taking to determine that potential participants have the capacity to participate in the decision-making process? If your study may enroll adults who are unable to consent, describe (i) how you will assess the capacity to consent, (ii) what provisions will be taken if the participant regains the capacity to consent,(iii) who will be used as a legally authorized representative, and (iv) what provisions will be made for the assent of the participant.**

Parents and legally authorized representatives will be allowed to make the determination of participation in this study. Parents will have the opportunity to ask additional questions about the study if they wish. We

Title : The Health and Wellness Curriculum Assessment
Approval Period: 04/24/2015 - 09/23/2015

will also discuss the study with the student participants and determine their understanding, and we will obtain written Assent after they have the opportunity to ask questions.

Select ALL applicable regulatory criteria for a Waiver of Documentation and provide a protocol-specific justification:

1) **45 CFR 46.117(c)(1)(i)., that the only record linking the participants and the research would be the consent document, and the principal risk would be potential harm resulting from a breach of confidentiality; each participant (or legally authorized representative) will be asked whether he/she wants documentation linking the participant with the research, and the participant's wishes govern.**

2) **45 CFR 46.117(c)(1)(ii)., that the research presents no more than minimal risk of harm to participants and involves no procedures for which written consent is normally required outside of the research context.**

3) **45 CFR 46.117(c)(1)(iii)., if participants or legally authorized representatives (LAR) are members of a distinct cultural group in which signing forms is not the norm, the research presents no more than minimal risk and there is an appropriate alternative mechanism for documenting that informed consent was obtained.**

4) Y **21 CFR 56.109(c)(1)., presents no more than minimal risk of harm to participants and involves no procedures for which written consent is normally required outside of the research context.**

Rationale for above selection:

For this portion of our study, which only involves answering some questionnaires about thoughts, feelings and academics, we request a Waiver of Documentation of Consent and Alteration of HIPPA Authorization because it is extremely difficult to secure a signature from parents in this population as many work more than one job, family life can be very unpredictable and many parents are not actively involved in school activities. The questionnaires present no more than minimal risk of harm. If a parent wishes to have his or her child opt out of the study, they may contact their principal or research study staff to notify them. Research study staff will follow up with as many parents as possible to complete the additional questionnaires (like Medical and Developmental History Form) covered through informed consent without signature. The original Questionnaire Consent will still be used to secure signatures from parents when possible, because we will need signed consent to access their child's academic records and to comply with FERPA.

13. 7 Consent   Comprehensive Consent (Eng)_FINAL_12.17.14

Check if VA related

a) **Describe the informed consent process. Include the following.**
- i) **Who is obtaining consent? (The person obtaining consent must be knowledgeable about the study.)**
- ii) **When and where will consent be obtained?**
- iii) **How much time will be devoted to consent discussion?**
- iv) **Will these periods provide sufficient opportunity for the participant to consider whether or not to participate and sign the written consent?**
- v) **What steps are you taking to minimize the possibility of coercion and undue influence?**
- vi) **If consent relates to children and if you have a reason for only one parent signing, provide that rationale for IRB consideration.**

Trained research staff will obtain consent from the parent at the child's school, home or other community setting prior to participation in any part of the study. Approximately 30-45 minutes will be devoted to consent discussion. Subjects will be told that participation is voluntary and that they may withdraw at any time.

b) **What is the Procedure to assess understanding of the information contained in the consent? How will the information be provided to participants if they do not understand English or if they have a hearing impairment? See HRPP Chapter12.2 for guidance.**

Research staff will ask questions about the components of the study to assess participants' understanding. Participants will also be given the opportunity to ask questions. English and Spanish translations of the consent will be provided. English and Spanish speaking research staff are available to discuss the consent with participants.

c) **What steps are you taking to determine that potential participants have the capacity to participate in the decision-making process? If your study may enroll adults who are unable to consent, describe (i) how you will assess the capacity to consent, (ii) what provisions will be taken if the participant regains**

the capacity to consent,(iii) who will be used as a legally authorized representative, and (iv) what provisions will be made for the assent of the participant.

Research staff will ask questions about the components of the study to assess participants' understanding. Participants will also be given the opportunity to ask questions. English and Spanish translations of the consent will be provided. English and Spanish speaking research staff are available to discuss the consent with participants. Research staff will also explain the study to the student and answer any questions he or she has. Signed Assent will also be obtained.

13. 8 Consent — Comprehensive Consent (Sp)_FINAL_12.17.14

Check if VA related

a) Describe the informed consent process. Include the following.
- **i) Who is obtaining consent? (The person obtaining consent must be knowledgeable about the study.)**
- **ii) When and where will consent be obtained?**
- **iii) How much time will be devoted to consent discussion?**
- **iv) Will these periods provide sufficient opportunity for the participant to consider whether or not to participate and sign the written consent?**
- **v) What steps are you taking to minimize the possibility of coercion and undue influence?**
- **vi) If consent relates to children and if you have a reason for only one parent signing, provide that rationale for IRB consideration.**

Trained research staff will obtain consent from the parent at the child's school, home or other community setting prior to participation in any part of the study. Approximately 30-45 minutes will be devoted to consent discussion. Subjects will be told that participation is voluntary and that they may withdraw at any time.

b) What is the Procedure to assess understanding of the information contained in the consent? How will the information be provided to participants if they do not understand English or if they have a hearing impairment? See HRPP Chapter12.2 for guidance.

Research staff will ask questions about the components of the study to assess participants' understanding. Participants will also be given the opportunity to ask questions. English and Spanish translations of the consent will be provided. English and Spanish speaking research staff are available to discuss the consent with participants.

c) What steps are you taking to determine that potential participants have the capacity to participate in the decision-making process? If your study may enroll adults who are unable to consent, describe (i) how you will assess the capacity to consent, (ii) what provisions will be taken if the participant regains the capacity to consent,(iii) who will be used as a legally authorized representative, and (iv) what provisions will be made for the assent of the participant.

Research staff will ask questions about the components of the study to assess participants' understanding. Participants will also be given the opportunity to ask questions. English and Spanish translations of the consent will be provided. English and Spanish speaking research staff are available to discuss the consent with participants. Research staff will also explain the study to the student and answer any questions he or she has. Signed Assent will also be obtained.

13. 9 Consent — Orchard School District Consent_English

Check if VA related

a) Describe the informed consent process. Include the following.
- **i) Who is obtaining consent? (The person obtaining consent must be knowledgeable about the study.)**
- **ii) When and where will consent be obtained?**
- **iii) How much time will be devoted to consent discussion?**
- **iv) Will these periods provide sufficient opportunity for the participant to consider whether or not to participate and sign the written consent?**
- **v) What steps are you taking to minimize the possibility of coercion and undue influence?**
- **vi) If consent relates to children and if you have a reason for only one parent signing, provide that rationale for IRB consideration.**

Consent will be obtained from parents by trained research staff listed on protocol. Consent will be obtained during one-on-one or group consultation (parent info session) about the study. Approximately 30 minutes will be devoted to consent discussion. Parents will be given time to ask questions and have all concerns addressed prior to providing written consent. Potential participants will be reminded that participation in the study is voluntary and they may discontinue their participation at any time without loss of education or

medical benefits to which they would otherwise be eligible to receive. The common family structure in this population often only includes one parent. Therefore, we are only requiring the signature of one parent for a child to participate in the study.

b) What is the Procedure to assess understanding of the information contained in the consent? How will the information be provided to participants if they do not understand English or if they have a hearing impairment? See HRPP Chapter12.2 for guidance.

Parents will be provided information in their preferred language or a translator will be provided. The research team will ask questions to assess parents' understanding and parents will be given time to ask questions.

c) What steps are you taking to determine that potential participants have the capacity to participate in the decision-making process? If your study may enroll adults who are unable to consent, describe (i) how you will assess the capacity to consent, (ii) what provisions will be taken if the participant regains the capacity to consent,(iii) who will be used as a legally authorized representative, and (iv) what provisions will be made for the assent of the participant.

The research team will ask questions to assess participants' understanding and participants will be given time to ask questions. We will obtain written assent from students.

13. 10 Consent — Orchard School District Consent_Spanish

Check if VA related

a) Describe the informed consent process. Include the following.

i) Who is obtaining consent? (The person obtaining consent must be knowledgeable about the study.)

ii) When and where will consent be obtained?

iii) How much time will be devoted to consent discussion?

iv) Will these periods provide sufficient opportunity for the participant to consider whether or not to participate and sign the written consent?

v) What steps are you taking to minimize the possibility of coercion and undue influence?

vi) If consent relates to children and if you have a reason for only one parent signing, provide that rationale for IRB consideration.

Consent will be obtained from parents by trained research staff listed on protocol. Consent will be obtained during one-on-one or group consultation (parent info session) about the study. Approximately 30 minutes will be devoted to consent discussion. Parents will be given time to ask questions and have all concerns addressed prior to providing written consent. Potential participants will be reminded that participation in the study is voluntary and they may discontinue their participation at any time without loss of education or medical benefits to which they would otherwise be eligible to receive. The common family structure in this population often only includes one parent. Therefore, we are only requiring the signature of one parent for a child to participate in the study.

b) What is the Procedure to assess understanding of the information contained in the consent? How will the information be provided to participants if they do not understand English or if they have a hearing impairment? See HRPP Chapter12.2 for guidance.

Parents will be provided information in their preferred language or a translator will be provided. The research team will ask questions to assess parents' understanding and parents will be given time to ask questions.

c) What steps are you taking to determine that potential participants have the capacity to participate in the decision-making process? If your study may enroll adults who are unable to consent, describe (i) how you will assess the capacity to consent, (ii) what provisions will be taken if the participant regains the capacity to consent,(iii) who will be used as a legally authorized representative, and (iv) what provisions will be made for the assent of the participant.

The research team will ask questions to assess participants' understanding and participants will be given time to ask questions. We will obtain written assent from students.

14. Assent Background (less than 18 years of age)

14. 1 Assent — HWC Child Assent (Revised)

a) Describe the assent process. Include the following:

(i) Who is obtaining child assent? (The person must be knowledgeable about the study.)

Title : The Health and Wellness Curriculum Assessment
Approval Period: 04/24/2015 - 09/23/2015

(ii) When and where will assent be obtained?
(iii) Will a parent or guardian be present when assent is obtained?
(iv) How much time will be devoted to the assent discussion?
(v) Will these periods provide sufficient opportunity for the child to consider whether to assent?
(vi) What steps are you taking to minimize the possibility of coercion and undue influence?

i. Assent will be obtained by trained research staff.
II. Assent will be obtained in the classroom at the beginning of the new curriculum.
iii. A parent or guardian will not be present, however the parents of children presented with an assent form will have already provided consent on their behalf.
iv. As much time as is needed will be devoted to the discussion of assent.
v. Yes.
vi. Emphasis will be placed on the voluntary nature of participation in this study. Students will be informed that there will be no consequences of any kind if they do not want to participate.

b) What is the procedure to assess the child's understanding of the information contained in the assent? How will the information be provided to the child if he/she does not understand English or has a hearing impairment? How will affirmative assent be obtained (e.g., oral response, signature on form, combination of methods, other)? Is there a possibility that the intervention or procedure involved in the research/clinical investigation holds out a prospect of direct benefit that is important to the health or well-being of the children and is available only in the context of the research/clinical investigation and therefore assent may not be necessary?

Research staff will be present with the participant and provide ample time to ask questions. If a child cannot read the form, research staff will read it to them. Child participants will be informed of the importance of asking questions if they do not understand.

c) What steps are you taking to determine that the child has the capacity to participate in the decision-making process?

During the assenting process, research staff will be working with teachers, school staff, para-educators, and principles to make sure that students who might need more support in understanding the assent form receive said support.

14. 2 Assent — Child Comprehensive Assent-Final

a) Describe the assent process. Include the following:

(i) Who is obtaining child assent? (The person must be knowledgeable about the study.)
(ii) When and where will assent be obtained?
(iii) Will a parent or guardian be present when assent is obtained?
(iv) How much time will be devoted to the assent discussion?
(v) Will these periods provide sufficient opportunity for the child to consider whether to assent?
(vi) What steps are you taking to minimize the possibility of coercion and undue influence?

Prior to participation in the study, research study staff will obtain child assent at the school and/or at child's home and/or Stanford campus depending on the component of the study. The parent will be present when assent is obtained. Researchers will discuss assent with the child for approximately 10-15 minutes (or for the length of time required to answer all of the child's questions about the study satisfactorily. Study staff will emphasize that participation is entirely voluntary and they can withdraw at any time without penalty.

b) What is the procedure to assess the child's understanding of the information contained in the assent? How will the information be provided to the child if he/she does not understand English or has a hearing impairment? How will affirmative assent be obtained (e.g., oral response, signature on form, combination of methods, other)? Is there a possibility that the intervention or procedure involved in the research/clinical investigation holds out a prospect of direct benefit that is important to the health or well-being of the children and is available only in the context of the research/clinical investigation and therefore assent may not be necessary?

Research study staff will discuss the study components with the child and answer any questions they have. Staff will also ask child questions to assess his/her understanding of the study and its requirements. All child participants in this study will be fluent in English. However, research study staff fluent in Spanish may also be present in the event there are issues with understanding.

c) What steps are you taking to determine that the child has the capacity to participate in the decision-making process?

Research study staff will discuss the study components with the child and answer any questions they have. Staff will also ask child questions to assess his/her understanding of the study and its requirements. Parents will be asked medical/developmental history questions to determine any issues concerning diagnoses (ex. learning disability). IRB has previously determined that consent must be obtained from only one parent based on the study population where it is often difficult or impossible to locate both parents for consent.

14. 3 Assent — Child Questionnaire Assent- Final

a) Describe the assent process. Include the following:

- **(i) Who is obtaining child assent?** (**The person must be knowledgeable about the study.**)
- **(ii) When and where will assent be obtained?**
- **(iii) Will a parent or guardian be present when assent is obtained?**
- **(iv) How much time will be devoted to the assent discussion?**
- **(v) Will these periods provide sufficient opportunity for the child to consider whether to assent?**
- **(vi) What steps are you taking to minimize the possibility of coercion and undue influence?**

Prior to participation in the study, research study staff will obtain child assent at the school and/or at child's home and/or Stanford campus depending on the component of the study. The parent may be present if assent is obtained at home or school but will not be required. Researchers will discuss assent with the child for approximately 10 minutes (or for the length of time required to answer all of the child's questions about the study satisfactorily. Study staff will emphasize that participation is entirely voluntary and they can withdraw at any time without penalty.

b) What is the procedure to assess the child's understanding of the information contained in the assent? How will the information be provided to the child if he/she does not understand English or has a hearing impairment? How will affirmative assent be obtained (e.g., oral response, signature on form, combination of methods, other)? Is there a possibility that the intervention or procedure involved in the research/clinical investigation holds out a prospect of direct benefit that is important to the health or well-being of the children and is available only in the context of the research/clinical investigation and therefore assent may not be necessary?

Research study staff will discuss the study components with the child and answer any questions they have. Staff will also ask child questions to assess his/her understanding of the study and its requirements. All child participants in this study will be fluent in English. However, research study staff fluent in Spanish may also be present in the event there are issues with understanding.

c) What steps are you taking to determine that the child has the capacity to participate in the decision-making process?

Research study staff will discuss the study components with the child and answer any questions they have. Staff will also ask child questions to assess his/her understanding of the study and its requirements. IRB has previously determined that consent must be obtained from only one parent based on the study population where it is often difficult or impossible to locate both parents for consent.

15. HIPAA Background

15. 1 Alteration of Authorization — aoa for questionnaire consent

a) Describe the Protected Health Information (PHI) needed to conduct the research. PHI is health information linked to HIPAA identifiers. List BOTH health information AND HIPAA identifiers. If you are using STRIDE, use the Data Privacy Attestation to ensure that your request will match your IRB-approved protocol.

PHI such as name, age, contact information and medical and demographic history will be obtained and deidentified using unique study IDs. This information will not be disclosed to others. Please see the attached Medical and Demographic History form for a complete list of the PHI being collected.

b) Please Answer:

Y **Do you certify that the use or disclosure of protected health information involves no more than a**

Title : The Health and Wellness Curriculum Assessment
Approval Period: 04/24/2015 - 09/23/2015

minimal risk to the privacy of individuals?

Y **Do you certify that the research could not practically be conducted with out the waiver?**

Y **Do you certify that you have adequate written assurances that the protected health information will not be reused or disclosed to any other person or entity, except as required by law, for authorized oversight of the research project, or for other research for which the use or disclosure of protected health information would be permitted?**

Y **Do you certify that the research could not practically be conducted with out access to and use of the protected health information?**

c) Please describe an adequate plan to protect any identifiers from improper use and disclosure.

For each subject, a folder will be created that contains all data for subject and family. Subject identity will be numerically coded on the subject folders and will be stored in a locked file cabinet accessible only to study personnel. Data will be entered into the computerized database using the subject ID number. Data will be labeled with each subject name linked to a unique numeric subject id. There will be a password protected computer file containing the master list. The de-identification will be performed by members of the research team.

d) Please describe an adequate plan to destroy the identifiers at the earliest opportunity consistent with conduct of the research, unless there is a health or research justification for retaining the identifiers or such retention is otherwise required by law.

Once subject data has been de-identified and the numeric subject ID assigned to each subject, material with identifiers (ex. consent forms) will be separated from the other data and kept in a separate locked cabinet in our lab at Psychiatry.

16. Attachments

Attachment Name	Attached Date	Attached By	Submitted Date
BASC-2 SRP Age 8-11	08/22/2014	awbasile	
BASC-2_PRS-A_age12-21	08/22/2014	awbasile	
BASC-2_PRS-A_age12-21_Sp	08/22/2014	awbasile	
BASC-2_PRS-C_age6-11	08/22/2014	awbasile	
BASC-2_PRS-C_age6-11_Sp	08/22/2014	awbasile	
BASC-2_SRP-A_age12-21	08/22/2014	awbasile	
Trauma Symptom Checklist for Children	08/25/2014	awbasile	
MRI Prescreening Form	08/25/2014	awbasile	
Childrens Sleep Habits Questionnaire	08/25/2014	awbasile	
BRIEF Parent Form	08/25/2014	awbasile	
BRIEF Teacher Form	08/25/2014	awbasile	
BRIEF-SR Rating Form	08/25/2014	awbasile	
WRAT4 Blue Response Form	08/25/2014	awbasile	

Title : The Health and Wellness Curriculum Assessment
Approval Period: 04/24/2015 - 09/23/2015

WRAT4 Blue Sentence Comprehension Test Form	08/25/2014	awbasile	
WRAT4 Blue Test Form	08/25/2014	awbasile	
WRAT4 Green Response Form	08/25/2014	awbasile	
WRAT4 Green Sentence Comprehension Test Form	08/25/2014	awbasile	
WRAT4 Green Test Form	08/25/2014	awbasile	
Medical and Demographic History Form	08/26/2014	awbasile	
Intial Contact Letter_Final Draft	08/26/2014	awbasile	
Proposal for Sonia and Paul Jones and the Sonima F	09/16/2014	awbasile	
Kaufman Assessment Battery for Children- II	09/18/2014	travisb	
Edinburg Handedness Form-English	09/18/2014	travisb	
Edinburg Handedness-Spanish	09/22/2014	travisb	
Medical and Demographic History Form (Spanish)	09/22/2014	awbasile	
Letter of Agreement	09/22/2014	jhowden	
Div Chief email	09/22/2014	jhowden	
scientific_scholarly_reviewAPP03010_Hardan	09/22/2014	jhowden	
Study Flyer_English	10/13/2014	travisb	
Study flyer_Spanish	10/13/2014	travisb	
Parent Retreat Handout	10/13/2014	travisb	
SCSNL Handedness Test-English	10/24/2014	travisb	
Tanner Stages Form- Male	10/24/2014	travisb	
Tanner Stages Form- Female	10/24/2014	travisb	
RSQ Student Survey	11/03/2014	travisb	
Child Sleep Habits Questionnaire	11/05/2014	travisb	
Morningness Eveningness Qustionnaire	11/05/2014	travisb	

Title : The Health and Wellness Curriculum Assessment
Approval Period: 04/24/2015 - 09/23/2015

Child Sleep Habits Questionnaire- Spanish Version	11/14/2014	travisb	
Impulsivity Scale for Children	11/14/2014	travisb	
Questionnaire Consent Parent Cover Letter	11/14/2014	travisb	
Questionnaire Consent Parent Letter- Spanish	11/17/2014	travisb	
Saliva Instructions & Questionnaires_Spanish_12.8	12/12/2014	travisb	
Saliva Instructions & Questionnaires_English_12.8	12/12/2014	travisb	
Info Brochure English	12/12/2014	travisb	
Info Brochure Spanish	12/12/2014	travisb	
H&W letter Parent questionnaires_Final_English	02/11/2015	travisb	
H&W Letter Parent Questionnaires_Final_Spanish	02/11/2015	travisb	
Orchard Approval Email	04/09/2015	travisb	

Obligations

The Protocol Director agrees to:

- Adhere to principles of sound scientific research designed to yield valid results
- Conduct the study according to the protocol approved by the IRB
- Be appropriately qualified to conduct the research and be trained in Human Research protection, ethical principles, regulations, policies and procedures
- Ensure all Stanford research personnel are adequately trained and supervised
- Ensure that the rights and welfare of participants are protected including privacy and confidentiality of data
- Ensure that, when de-identified materials are obtained for research purposes, no attempt will be made to re-identify them.
- Disclose to the appropriate entities any potential conflict of interest
- Report promptly any new information, modification, or unanticipated problems that raise risks to participants or others
- Apply relevant professional standards.

Any change in the research protocol must be submitted to the IRB for review prior to the implementation of such change. Any complications in participants or evidence of increase in the original estimate of risk should be reported at once to the IRB before continuing with the project. Inasmuch as the Institutional Review Board (IRB) includes faculty, staff, legal counsel, public members, and students, protocols should be written in language that can be understood by all Panel members. The investigators must inform the participants of

Title : The Health and Wellness Curriculum Assessment
Approval Period: 04/24/2015 - 09/23/2015

any significant new knowledge obtained during the course of the research.

IRB approval of any project is for a maximum period of one year. For continuing projects and activities, it is the responsibility of the investigator(s) to resubmit the project to the IRB for review and re-approval prior to the end of the approval period. A Notice to Renew Protocol is sent to the Protocol Director 7 weeks prior to the expiration date of the protocol.

https://stanfordmedicine.box.com/shared/static/qbsi8u8h47qsotxhdpuzz50xlrqa0sgo.pdf Report promptly any new information, complaints, possibly serious and/or continuing noncompliance, or unanticipated problems involving risks to participants or others.

All data including signed consent form documents must be retained for a minimum of three years past the completion of the research. Additional requirements may be imposed by your funding agency, your department, or other entities. (Policy on Retention of and Access to Research Data, Research Policy Handbook, http://doresearch.stanford.edu/policies/research-policy-handbook/conduct-research/retention-and-access-research-data)

APPROVAL LETTER/NOTICE NOTE: List all items (verbatim) that you want to be included in your approval letter (e.g., Amendment date, Investigator's Brochure version, consent form(s) version(s), advertisement name, etc.) in the box below.

Updated information about control group recruitment and assessment
Consent form for control group (English and Spanish versions)
Tentative agreement/partnership email from control school district

Y By checking this box, I verify that I, as the Protocol Director (PD) responsible for this research protocol, have read and agree to abide by the above obligations, or that I have been delegated authority by the PD to certify that the PD has read and agrees to abide by the above obligations.

Comments

Comment Title	Comments / Responses	Response Necessary
**REVISION : 04/28/2015**		
**Cycle: 1**		
1	Please update sections 8(a) and 8(g) to reflect the changes to the study population.<br>Updated sections 8(a) and 8(g) to include control students from Orchard School District.	Y
