# Supplementary material for: Program evaluation of a school-based mental health and wellness curriculum featuring yoga and mindfulness
Source: PLoS One. 2024 Apr 4;19(4):e0301028. doi: 10.1371/journal.pone.0301028 (PMC10994323; doi:10.1371/journal.pone.0301028)
Supplement: S1 Table — (DOCX) [file pone.0301028.s003.docx]

| **Table S1. Demographic Distributions by Study Group and Paired Schools (% at Time 1).** | | | | | | | | |
| --- | --- | --- | --- | --- | --- | --- | --- | --- |
|  | Pair A | | Pair B | | Pair C | | Pair D | |
|  | Ctrl. | Int. | Ctrl. | Int. | Ctrl. | Int. | Ctrl. | Int. |
| *Gender* |  | | | | | | | |
| Male | 56.1 | 43.9 | 51.6 | 47.3 | 49.5 | 46.2 | 39.1 | 51.2 |
| Female | 43.9 | 55.0 | 46.9 | 52.7 | 50.5 | 53.8 | 57.9 | 48.8 |
| Missing | 0.0 | 1.2 | 1.6 | 0.0 | 0.0 | 0.0 | 3.0 | 0.0 |
| *Age* |  | | | | | | | |
| 7 | 0.0 | 0.0 | 0.0 | 0.0 | 0.0 | 0.0 | 0.8 | 0.0 |
| 8 | 41.5 | 37.4 | 39.1 | 69.1 | 39.2 | 41.5 | 40.6 | 40.3 |
| 9 | 5.7 | 9.9 | 3.1 | 3.6 | 3.1 | 5.7 | 6.0 | 5.4 |
| 10 | 48.0 | 37.4 | 42.2 | 20.0 | 51.5 | 25.5 | 46.6 | 34.1 |
| 11 | 3.3 | 10.5 | 9.4 | 3.6 | 5.2 | 16.0 | 3.8 | 11.6 |
| 12 | 0.0 | 1.8 | 0.0 | 1.8 | 0.0 | 0.0 | 0.0 | 0.0 |
| 13 | 0.0 | 0.6 | 0.0 | 0.0 | 0.0 | 0.0 | 0.0 | 0.0 |
| Missing | 1.6 | 2.3 | 6.3 | 1.8 | 1.0 | 11.3 | 2.3 | 8.5 |
| *Race/Ethnicity* |  | | | | | | | |
| American Indian or Alaska Native | 20.3 | 0.6 | 10.9 | 1.8 | 9.3 | 0.0 | 8.3 | 0.8 |
| Chinese | 0.8 | 0.0 | 0.0 | 0.0 | 0.0 | 0.0 | 0.0 | 0.0 |
| Vietnamese | 6.5 | 0.0 | 1.6 | 0.0 | 5.2 | 0.0 | 7.5 | 0.0 |
| Asian Indian | 0.8 | 0.6 | 0.0 | 0.0 | 0.0 | 0.0 | 0.8 | 0.0 |
| Laotian | 0.0 | 0.0 | 0.0 | 0.0 | 0.0 | 0.0 | 0.8 | 0.0 |
| Cambodian | 1.6 | 0.0 | 1.6 | 0.0 | 1.0 | 0.0 | 0.0 | 0.0 |
| Other Asian | 0.8 | 0.6 | 3.1 | 0.0 | 7.2 | 0.0 | 2.3 | 0.0 |
| Guamanian | 0.0 | 0.0 | 0.0 | 0.0 | 0.0 | 0.0 | 0.8 | 0.0 |
| Samoan | 0.0 | 1.8 | 0.0 | 1.8 | 0.0 | 0.9 | 1.5 | 7.8 |
| Other Pacific Islander | 0.8 | 1.8 | 0.0 | 0.0 | 1.0 | 3.8 | 0.8 | 4.7 |
| Filipino | 0.8 | 0.0 | 1.6 | 0.0 | 1.0 | 0.0 | 4.5 | 0.0 |
| Hispanic | 55.3 | 57.9 | 73.4 | 85.5 | 72.2 | 64.2 | 54.1 | 52.7 |
| Black | 0.0 | 9.4 | 1.6 | 0.0 | 0.0 | 7.5 | 4.5 | 8.5 |
| White | 4.1 | 0.0 | 1.6 | 0.0 | 1.0 | 0.0 | 3.8 | 0.0 |
| Missing | 8.1 | 27.5 | 4.7 | 10.9 | 2.1 | 23.6 | 10.5 | 25.6 |
| *Note*. Ctrl. = Control Group, Int. = Intervention Group, Details on N’s for each cell at each time point are provided in the supplemental tables S1-S5. | | | | | | | | |
