# Supplementary material for: Program evaluation of a school-based mental health and wellness curriculum featuring yoga and mindfulness
Source: PLoS One. 2024 Apr 4;19(4):e0301028. doi: 10.1371/journal.pone.0301028 (PMC10994323; doi:10.1371/journal.pone.0301028)
Supplement: S2 Table — (DOCX) [file pone.0301028.s004.docx]

| **Table S2. Means, Standard Deviations, and Effect Size Estimates for RSQ Primary Control Scores.** | | | | | | | |  |
| --- | --- | --- | --- | --- | --- | --- | --- | --- |
| **Measure** | **Group** | **Time 1** | **Time 2** | **Time 3** | **Effect size estimates** | | | |
|  |  | Mean(SD) | Mean(SD) | Mean(SD) | Time 1 to Time 2 | Time 2 to Time 3 | Time 1 to Time 3 | |
| ***Primary Control Composite*** |  |  |  |  |  |  |  | |
|  | Control | 0.91(0.42) n = 398 | 0.95(0.37) n = 289 | 0.99(0.40) n = 285 | -0.70 n = 277 | -0.06 n = 249 | -0.14 n = 274 | |
|  | Treatment^a,c^ | 0.88(0.41) n = 430 | 0.95(0.38) n = 352 | 0.97(0.39) n = 328 | -0.13 n = 331 | -0.07 n = 310 | -0.15 n = 307 | |
| ***Match A*** |  |  |  |  |  |  |  | |
|  | Control^a,c^ | 0.88(0.40) n = 120 | 0.99(0.35) n = 83 | 1.09(0.41) n = 71 | -0.24 n = 81 | -0.20 n = 67 | -0.38 n = 69 | |
|  | Treatment^b^ | 0.95(0.39) n = 163 | 0.93(0.38) n = 130 | 0.97(0.38) n = 119 | 0.05  n = 126 | -0.16 n = 116 | -0.10 n = 115 | |
| ***Match B*** |  |  |  |  |  |  |  | |
|  | Control | 1.01(0.40) n = 58 | 0.91(0.43) n = 36 | 0.97(0.39) n = 38 | 0.23 n = 33 | -0.16 n = 28 | -0.01 n = 36 | |
|  | Treatment | 0.81(0.51) n = 48 | 0.86(0.36) n = 49 | 0.97(0.40) n = 46 | -0.06 n = 42 | -0.22 n = 42 | -0.22 n = 39 | |
| ***Match C*** |  |  |  |  |  |  |  | |
|  | Control^c^ | 0.85(0.39) n = 91 | 0.94(0.37) n = 79 | 0.98(0.43) n = 73 | -0.15 n = 74 | -0.10 n = 71 | -0.24 n = 68 | |
|  | Treatment | 0.93(0.42) n = 96 | 1.02(0.40) n = 79 | 1.02(0.42) n = 75 | -0.16 n = 73 | -0.06 n = 71 | -0.11 n = 69 | |
| ***Match D*** |  |  |  |  |  |  |  | |
|  | Control | 0.93(0.45) n = 129 | 0.95(0.36) n = 91 | 0.94(0.35) n = 103 | 0.02 n = 89 | 0.14 n = 83 | 0.03 n = 101 | |
|  | Treatment^a,c^ | 0.78(0.38) n = 123 | 0.96(0.35) n = 94 | 0.91(0.36) n = 88 | -0.39 n = 90 | 0.16 n = 81 | -0.21 n = 84 | |
|  |  |  |  |  |  |  |  | |
| **Emotion Regulation** |  |  |  |  |  |  |  | |
|  | Control | 0.89(0.50)  n = 392 | 0.88(0.48) n = 288 | 0.89(0.49) n = 285 | 0.04 n = 271 | 0.04 n = 248 | 0.03 n = 270 | |
|  | Treatment^a,c^ | 0.74(0.49) n = 429 | 0.84(0.48) n = 347 | 0.89(0.48) n = 328 | -0.13 n = 327 | -0.10 n = 305 | -0.21 n = 306 | |
| **Emotion Expression** |  |  |  |  |  |  |  | |
|  | Control^c^ | 0.84(0.49) n = 392 | 0.90(0.47) n = 289 | 0.93(0.51) n = 285 | -0.07 n = 275 | -0.03 n = 249 | -0.14 n = 269 | |
|  | Treatment^a,c^ | 0.86(0.52) n = 429 | 0.94(0.48) n = 350 | 0.96(0.49) n = 328 | -0.13 n = 329 | -0.04 n = 308 | -0.13 n = 206 | |
| ***Match A*** |  |  |  |  |  |  |  | |
|  | Control | 0.86(0.48) n = 120 | 0.93(0.47) n = 83 | 0.97(0.52) n = 71 | -0.11 n = 81 | -0.06 n = 67 | -0.18 n = 69 | |
|  | Treatment^b^ | 0.94(0.51) n = 163 | 0.90(0.47) n = 129 | 0.98(0.46) n = 119 | -0.10 n = 125 | -0.21 n = 115 | -0.07 n = 115 | |
| ***Match B*** |  |  |  |  |  |  |  | |
|  | Control^a^ | 1.00(0.46) n = 57 | 0.81(0.49) n = 36 | 0.96(0.53) n = 38 | 0.42 n = 33 | -0.17 n = 28 | -0.02 n = 36 | |
|  | Treatment^a,c^ | 0.68(0.49) n = 47 | 0.97(0.49) n = 48 | 0.97(0.62) n = 46 | -0.45 n = 41 | 0.05 n = 41 | -0.34 n = 38 | |
| ***Match C*** |  |  |  |  |  |  |  | |
|  | Control | 0.78(0.48) n = 90 | 0.91(0.46) n = 79 | 0.90(0.58) n = 73 | -0.17 n = 73 | 0.00 n = 71 | -0.16 n = 67 | |
|  | Treatment | 0.91(0.51) n = 96 | 0.99(0.52) n = 79 | 1.03(0.50) n = 75 | -0.05 n = 73 | -0.10 n = 71 | -0.03 n = 69 | |
| ***Match D*** |  |  |  |  |  |  |  | |
|  | Control | 0.79(0.51) n = 125 | 0.89(0.47) n = 91 | 0.92(0.45) n = 103 | -0.12 n = 88 | 0.03 n = 83 | -0.14 n = 97 | |
|  | Treatment^a,b,c^ | 0.76(0.52) n = 123 | 0.95(0.45) n = 94 | 0.87(0.43) n = 88 | -0.36 n = 90 | 0.24 n = 81 | -0.21 n = 84 | |
| *Note*. ^a^ indicates a significant change from Time 1 to Time 2, ^b^ indicates a significant change from Time 2 to Time 3, and ^c^ indicates a significant change from Time 1 to Time 3. | | | | | | | | |
