# Supplementary material for: Program evaluation of a school-based mental health and wellness curriculum featuring yoga and mindfulness
Source: PLoS One. 2024 Apr 4;19(4):e0301028. doi: 10.1371/journal.pone.0301028 (PMC10994323; doi:10.1371/journal.pone.0301028)
Supplement: S3 Table — (DOCX) [file pone.0301028.s005.docx]

| **Table S4. Summary of ANOVAs.** Effects of time, study group, and matched pair on RSQ Involuntary Engagement and subscales. | | | |
| --- | --- | --- | --- |
| Effect | *F* | *df* | *p* |
| Involuntary Engagement Composite | | | |
| Time | .68 | 1.94, 1013.61 | .50 |
| Time x Study Group | .82 | 1.94, 1013.61 | .44 |
| Time x Matched Pair | 2.10 | 5.83, 1013.61 | .05† |
| Time x Study Group x Matched Pair | .55 | 5.83, 1013.61 | .77 |
| Rumination | | | |
| Time | 4.00 | 2, 1030 | < .05 |
| Time x Study Group | 1.32 | 2, 1030 | .32 |
| Time x Matched Pair | .51 | 6, 1030 | .80 |
| Time x Study Group x Matched Pair | .72 | 6, 1030 | .64 |
| Intrusive Thoughts | | | |
| Time | .01 | 1.98, 1029.53 | .99 |
| Time x Study Group | .48 | 1.98, 1029.53 | .42 |
| Time x Matched Pair | 1.56 | 5.93, 1029.53 | .56 |
| Time x Study Group x Matched Pair | .01 | 5.93, 1029.53 | .99 |
| Physiological Arousal | | | |
| Time | 4.37 | 1.97, 1013.99 | < .05 |
| Time x Study Group | 1.51 | 1.97, 1013.99 | .22 |
| Time x Matched Pair | 2.70 | 5.90, 1013.99 | < .05 |
| Time x Study Group x Matched Pair | .66 | 5.90, 1013.99 | .68 |
| Emotional Arousal | | | |
| Time | .22 | 1.95, 1013.33 | .80 |
| Time x Study Group | 1.45 | 1.95, 1013.33 | .24 |
| Time x Matched Pair | 2.31 | 5.86, 1013.33 | < .05 |
| Time x Study Group x Matched Pair | .55 | 5.86, 1013.33 | .76 |
| Involuntary Action | | | |
| Time | 1.36 | 1.97, 1026.71 | .26 |
| Time x Study Group | 1.38 | 1.97, 1026.71 | .25 |
| Time x Matched Pair | 1.31 | 5.92, 1026.71 | .25 |
| Time x Study Group x Matched Pair | .69 | 5.92, 1026.71 | .66 |
| *Note*. † indicates trending significance | | | |
