# Supplementary material for: Program evaluation of a school-based mental health and wellness curriculum featuring yoga and mindfulness
Source: PLoS One. 2024 Apr 4;19(4):e0301028. doi: 10.1371/journal.pone.0301028 (PMC10994323; doi:10.1371/journal.pone.0301028)
Supplement: S5 Table — Effects of time, study group, and matched pair on BASC scale scores. (DOCX) [file pone.0301028.s007.docx]

| **Table S5. Summary of ANOVAs.** Effects of time, study group, and matched pair on BASC scale scores. | | | |
| --- | --- | --- | --- |
| Effect | *F* | *df* | *p* |
| Anxiety | | | |
| Time | 5.15 | 1.96, 1020.53 | < .01 |
| Time x Study Group | .70 | 1.96, 1020.53 | .49 |
| Time x Matched Pair | .81 | 5.88, 1020.53 | .56 |
| Time x Study Group x Matched Pair | .53 | 5.88, 1020.53 | .78 |
| Depression | | | |
| Time | 21.99 | 2, 1042 | < .001 |
| Time x Study Group | 2.65 | 2, 1042 | .07† |
| Time x Matched Pair | .65 | 6, 1042 | .69 |
| Time x Study Group x Matched Pair | 1.15 | 6, 1042 | .33 |
| Attention Problems | | | |
| Time | .48 | 1.93, 1003.74 | .61 |
| Time x Study Group | 1.64 | 1.93, 1003.74 | .20 |
| Time x Matched Pair | .38 | 5.78, 1003.74 | .89 |
| Time x Study Group x Matched Pair | .82 | 5.78, 1003.74 | .55 |
| Hyperactivity | | | |
| Time | .17 | 1.96, 1018.64 | .84 |
| Time x Study Group | .91 | 1.96, 1018.64 | .40 |
| Time x Matched Pair | .42 | 5.87, 1018.64 | .86 |
| Time x Study Group x Matched Pair | .71 | 5.87, 1018.64 | .64 |
| *Note*. † indicates trending significance | | | |
