# Supplementary material for: Program evaluation of a school-based mental health and wellness curriculum featuring yoga and mindfulness
Source: PLoS One. 2024 Apr 4;19(4):e0301028. doi: 10.1371/journal.pone.0301028 (PMC10994323; doi:10.1371/journal.pone.0301028)
Supplement: S6 Table — (DOCX) [file pone.0301028.s008.docx]

| **Table S6. Means, Standard Deviations, and Effect Size on BASC Scores.** | | | | | | | |
| --- | --- | --- | --- | --- | --- | --- | --- |
| **Measure** | **Group** | **Time 1** | **Time 2** | **Time 3** | **Effect size estimates** | | |
|  |  | Mean(SD) | Mean(SD) | Mean(SD) | Time 1 to Time 2 | Time 2 to Time 3 | Time 1 to Time 3 |
| ***BASC Subscales*** |  |  |  |  |  |  |  |
| **Anxiety** |  |  |  |  |  |  |  |
|  | Control^b^ | 14.29(6.71) n = 404 | 15.07(7.39) n = 284 | 13.83(7.37) n = 281 | -0.07 n = 275 | 0.22 n = 242 | 0.09 n = 274 |
|  | Treatment^b^ | 13.68(6.65) n = 428 | 14.36(7.48) n = 346 | 13.30(6.94) n = 328 | -0.08 n = 331 | 0.11 n = 307 | 0.05 n = 314 |
| **Depression** |  |  |  |  |  |  |  |
|  | Control^a,b,c^ | 9.55(6.49) n = 404 | 9.00(7.24) n = 284 | 7.23(7.07) n = 281 | 0.12 n = 275 | 0.29 n = 242 | 0.32 n = 274 |
|  | Treatment^b,c^ | 8.40(6.11) n = 438 | 8.51(6.93) n = 346 | 7.23(6.76) n = 328 | 0.01 n = 331 | 0.17 n = 307 | 0.19 n = 314 |
| **Attention Problems** |  |  |  |  |  |  |  |
|  | Control^c^ | 4.50(4.07) n = 404 | 5.02(4.37) n = 284 | 5.91(4.75) n = 281 | 0.04 n = 275 | -0.12 n = 242 | -0.20 n = 274 |
|  | Treatment^a,b,c^ | 4.34(3.84) n = 438 | 5.96(5.07) n = 346 | 6.56(5.95) n = 328 | -0.29 n = 331 | -0.13 n = 307 | -0.33 n = 314 |
| **Hyperactivity** |  |  |  |  |  |  |  |
|  | Control | 7.15(4.21) n = 404 | 7.30(4.67) n = 284 | 7.09(4.17) n = 281 | -0.00 n = 275 | 0.10 n = 242 | 0.05 n = 274 |
|  | Treatment | 6.87(4.34) n = 438 | 7.13(4.23) n = 346 | 7.07(4.14) n = 328 | -0.04 n = 331 | -0.02 n = 307 | -0.03 n = 314 |
| *Note*. ^a^ indicates a significant change from Time 1 to Time 2, ^b^ indicates a significant change from Time 2 to Time 3, and ^c^ indicates a significant change from Time 1 to Time 3. | | | | | | | |
