# Supplementary material for: Program evaluation of a school-based mental health and wellness curriculum featuring yoga and mindfulness
Source: PLoS One. 2024 Apr 4;19(4):e0301028. doi: 10.1371/journal.pone.0301028 (PMC10994323; doi:10.1371/journal.pone.0301028)
Supplement: S7 Table — (DOCX) [file pone.0301028.s009.docx]

| **Table S7. Means, Standard Deviations, and Effect Size Estimates on RSQ Involuntary Engagement Scores.** | | | | | | | |  |
| --- | --- | --- | --- | --- | --- | --- | --- | --- |
| **Measure** | **Group** | **Time 1** | **Time 2** | **Time 3** | **Effect size estimates** | | | |
|  |  | Mean(SD) | Mean(SD) | Mean(SD) | Time 1 to Time 2 | Time 2 to Time 3 | Time 1 to Time 3 | |
| ***RSQ Subscales*** |  |  |  |  |  |  |  | |
| **Involuntary Engagement Composite** |  |  |  |  |  |  |  | |
|  | Control | 0.67(0.41) n = 398 | 0.68(0.42) n = 289 | 0.65(0.47) n = 285 | 0.02 n = 277 | 0.10 n = 249 | 0.05 n = 274 | |
|  | Treatment | 0.60(0.42) n = 430 | 0.63(0.41) n = 352 | 0.61(0.41) n = 328 | -0.09 n = 331 | 0.03 n = 310 | -0.04 n = 307 | |
| **Rumination** |  |  |  |  |  |  |  | |
|  | Control^b^ | 0.73(0.55) n = 387 | 0.76(0.56) n = 289 | 0.68(0.59) n = 285 | -0.03 n = 271 | 0.15 n = 249 | 0.05 n = 265 | |
|  | Treatment^a,b,c^ | 0.59(0.50) n = 429 | 0.71(0.55) n = 350 | 0.63(0.55) n = 327 | -0.19 n = 329 | 0.10 n = 308 | -0.10 n = 305 | |
| **Intrusive Thoughts** |  |  |  |  |  |  |  | |
|  | Control | 0.58(0.50) n = 395 | 0.56(0.46) n = 289 | 0.59(0.56) n = 285 | 0.03 n = 275 | -0.01 n = 249 | 0.03 n = 272 | |
|  | Treatment | 0.48(0.48) n = 429 | 0.52(0.50) n = 351 | 0.47(0.46) n = 328 | -0.07 n = 330 | 0.05 n = 310 | 0.01 n = 306 | |
| **Physiological Arousal** |  |  |  |  |  |  |  | |
|  | Control^c^ | 0.51(0.46) n = 394 | 0.47(0.47) n = 288 | 0.45(0.51) n = 285 | 0.09 n = 273 | 0.09 n = 248 | 0.13 n = 270 | |
|  | Treatment | 0.45(0.47) n = 429 | 0.48(0.48) n = 350 | 0.45(0.45) n = 327 | -0.01 n = 329 | 0.01 n = 308 | -0.06 n = 305 | |
| **Emotional Arousal** |  |  |  |  |  |  |  | |
|  | Control | 0.72(0.52) n = 396 | 0.73(0.52) n = 289 | 0.70(0.58) n = 285 | -0.00 n = 275 | 0.10 n = 249 | 0.04 n = 272 | |
|  | Treatment | 0.63(0.51) n = 429 | 0.67(0.49) n = 351 | 0.66(0.52) n = 328 | -0.07 n = 330 | -0.00 n = 309 | -0.07 n = 306 | |
| **Involuntary Action** |  |  |  |  |  |  |  | |
|  | Control | 0.76(0.55) n = 394 | 0.79(0.60) n = 289 | 0.76(0.61) n = 285 | 0.00 n = 273 | 0.02 n = 249 | -0.03 n = 272 | |
|  | Treatment^a,c^ | 0.67(0.57) n = 429 | 0.79(0.60) n = 352 | 0.72(0.55) n = 328 | -0.15* n = 331 | 0.02 n = 310 | -0.10* n = 306 | |
| *Note*. ^a^ indicates a significant change from Time 1 to Time 2, ^b^ indicates a significant change from Time 2 to Time 3, and ^c^ indicates a significant change from Time 1 to Time 3. | | | | | | | | |
