# Supplementary material for: Program evaluation of a school-based mental health and wellness curriculum featuring yoga and mindfulness
Source: PLoS One. 2024 Apr 4;19(4):e0301028. doi: 10.1371/journal.pone.0301028 (PMC10994323; doi:10.1371/journal.pone.0301028)
Supplement: Attachment — Submitted filename: Response to Review.docx [file pone.0301028.s010.docx]

Response to Reviewers

July 11, 2023

PLOS ONE (PONE-D-23-14991)

Dear Editors,

Thank you for the opportunity to resubmit our manuscript titled “Program Evaluation of a School-Based Mental Health and Wellness Curriculum Featuring Yoga and Mindfulness.” Below is the comment received by the Editorial Office and our response.

*“Dear Dr. McCurdy,*

*Thank you very much for submitting your manuscript to PLOS ONE, and for responding to our recent requests regarding your submission. After careful evaluation, we have decided that your submission does not meet our publication criteria and must be rejected.*

*Based on our evaluation of your manuscript and the information you provided, we do not feel that your submission meets our ethical requirements for human subjects research submissions. PLOS ONE requires that research meets all applicable standards for the ethics of experimentation and research integrity (http://journals.plos.org/plosone/s/human-subjects-research). We reserve the right to reject any submission that does not meet our internal ethical standards, which in some cases are more stringent than local ethical standards.*

*We recognize that you obtained ethical approval from your local ethics committee after you began the study: your ethics approval document is dated August 31, 2022, while participants were recruited beginning in 2014. For human subjects research, PLOS ONE requires that authors obtain ethics committee approval before beginning the study, and we do not accept retrospective ethical approval.*

*As a result of these concerns we cannot consider the manuscript for publication at PLOS ONE. I am sorry that we cannot be more positive on this occasion, but hope that you understand the reasons for this decision.”*

We thank you for your careful consideration of our manuscript and the ethical requirements needed for human subjects research. We apologize for the miscommunication in our original submission. When asked to include an IRB determination letter, we included the current IRB determination letter for *continuing review*. We understand that the *continuing review* approval was unclear regarding the time frame for the beginning of the study, as the letter was dated 2022. In our new submission, we are including the original IRB determination letter, dated 2014. We ensure that we received approval from our ethics committee prior to the beginning of the study and we did not receive retrospective ethical approval.

We sincerely thank you for your time and patience during our submission process. We appreciate the opportunity to submit our manuscript to PLOS ONE and look forward to hearing from you.

With gratitude,

The Authors
