# Supplementary material for: Program evaluation of a school-based mental health and wellness curriculum featuring yoga and mindfulness
Source: PLoS One. 2024 Apr 4;19(4):e0301028. doi: 10.1371/journal.pone.0301028 (PMC10994323; doi:10.1371/journal.pone.0301028)
Supplement: Attachment — Submitted filename: response letter PLOS One.docx [file pone.0301028.s011.docx]

Reviewer Response Letter

November 20, 2023

**Avanti Dey 
Staff Editor**

Dear Dr. Dey,

We extend our gratitude for inviting a second revision of our paper titled, “Program Evaluation of a School-Based Mental Health and Wellness Curriculum Featuring Yoga and Mindfulness” and for the thoughtful comments we received from the current reviewer. The review we have received has improved the quality of our manuscript. We have addressed each comment in the revised manuscript or in this response letter. In the following, we detail how we addressed each point in the order in which they appeared in your letter. We include the reviewer’s comments followed by our response to the comment in italicized font with page numbers as appropriate.

COMMENTS FOR THE AUTHOR:

**Reviewer #1**: Overall, this manuscript is clear, well-organized and written and offers important information about the challenges of program implementation. The authors are thorough in their reporting of the study justification, design and procedures. I have detailed specific comments below. I wonder if there could be more commentary on the challenges associated with program implementation since this could shed more light on study findings and be informative for readers.

Specific Comments:

• Overall, introduction is very nicely written and supported with relevant research. The background moves nicely from the problem to coping to mindfulness to yoga and the importance of fidelity and study design.

*We thank the reviewer very much for their constructive and encouraging comments on our introduction. With their feedback, we will ensure that this positive momentum is maintained throughout the entire manuscript.*

• P.10, bottom of first full paragraph – is nonattachment the only pathway through which mindfulness is theorized or shown to aid in emotion regulation?

*We thank the reviewer for pointing out this oversight. We have included more examples (with a new citation) of pathways through which mindfulness is thought to enhance solution-focused strategies and emotion regulation, such as through promoting goal orientation, problem disengagement, and mobilizing resources to focus on acceptance and awareness [page 10 of pdf, page 4 of manuscript]. This sentence now reads: “Effectively, mindfulness is thought to enhance the ability to engage in solution-focused strategies and emotion regulation through promoting goal orientation, problem disengagement, and mobilizing resources to focus on acceptance and awareness while minimizing avoidance-oriented coping by fostering an attitude of non-attachment towards stressful life experiences [15, 16].”*

• Bottom of p.10 – can the age ranges of the samples being discussed here be specified?

*Thanks for this suggestion. We double-checked and the specific ages or ranges were not reported for all papers; however, the grade ranges of youth in studies cited here are available and are now referenced in the text [page 10-11 of pdf, page 4-5 of manuscript].This sentence now reads: “Preliminary evidence shows some promise, as youth in second grade to twelfth grade who engage in yoga to promote mindfulness show improved coping skills, increased socio-emotional competence and prosocial skills, decreased alcohol use, and teacher-rated improvements in academic performance, attention span, and ability to deal with stress and feelings of anxiety [20-24].”*

• Top of p.11: “internalizing scores” may require some explanation.

*We have edited this sentence so it now reads: “For example, a multi-year longitudinal study [25] measuring the effectiveness of a mindfulness-based stress reduction intervention in 12–18-year-olds found that youth significantly improved their internalizing problems (i.e., anxiety, depression, and somatization) as measured by the Behavioral Assessment System for Children.” [page 11 of pdf, page 5 of manuscript]*

• In the discussion, I wonder if the authors could provide more contextual information about the challenges with implementation – factors that could have affected the results and more suggestions for future research. This seems to be the most valuable part of this study. I also wonder if there needs to be more discussion on why so few findings were significant as expected.

*We thank the reviewer for this suggestion, as the logistics of delivering this program in school settings, sustainability, and challenges when conducting a program evaluation are critical points made within this manuscript that address a strong need for consideration in future programming and evaluation. We believe the challenges in implementation attributed to mixed findings. To elaborate on these points, we have added the following text [page 22 of pdf, page 16 of manuscript]:*

*“Schools demonstrated variability in the fidelity of intervention implementation. For example, unforeseen circumstances had an impact on curriculum delivery, such as cancellation of classes for health-related reasons, variability of support from administration and teachers at individual schools, variability in how schools managed behavioral concerns (e.g., sending students out of class instead of keeping them in class), and the necessity to at times use substitute instructors, such as in the case of illness or injury, of which who may not have had the same experience level and rapport with students as the regular teacher. Program evaluation of these types of programs need to be flexible in fitting into the routine and the unforeseen vicissitudes of the school context [7], such as variability in staff support and facilities, as well as the complex logistics of delivering such programming [29].”*

*Note that we included a duplicate reference in the manuscript and the duplicate is now removed.*

In sum, we have considered each suggestion and have addressed each point. We once again

thank you for your time and close read of our manuscript. We look forward to hearing from you.

Sincerely,

The Authors
