# Supplementary material for: Program evaluation of a school-based mental health and wellness curriculum featuring yoga and mindfulness
Source: PLoS One. 2024 Apr 4;19(4):e0301028. doi: 10.1371/journal.pone.0301028 (PMC10994323; doi:10.1371/journal.pone.0301028)
Supplement: Attachment — Submitted filename: Response to Reviewers 2_22_2024.docx [file pone.0301028.s012.docx]

February 21, 2024	Response to Reviewers

**Annesha Sil**

**Associate Editor**

RE: PONE-D-23-21703R1

Program Evaluation of a School-Based Mental Health and Wellness Curriculum Featuring Yoga and Mindfulness

PLOS ONE

Dear Dr. Sil,

Thanks so much for your decision requesting additional minor revisions. One reviewer indicated that we had addressed all concerns and one reviewer outlined outstanding minor concerns, which we have addressed in our response below (in italics). We thank all reviewers again for the excellent feedback to improve the paper.

Reviewer 1: Had no additional comments and confirmed our responsiveness to the previous comments. We again thank this reviewer for the helpful comments to strengthen our paper.

Reviewer 2:

Although this is a Revision, I think the statistical analysis has been presented half-heartedly, and would require further work for improvement. I state those below.

*We first thank the reviewer for the suggestions for improvement below and address each point. We do disagree about our analysis presentation as being “half-hearted”. The supplemental information contains a plethora of statistical and descriptive data and some information on testing assumptions was actually in the previous draft (see below).*

1. Abstract: In the Results, statements of direction ("relative improvement on measures of emotion....") should be accompanied by a p-value.

*We wrote this paper using guidance supplied by the American Psychological Association (APA) and Journal of the American Medical Association (JAMA). APA style does not require p-values in abstracts. Indeed, many writing guides suggest to not include: “****Note****: Generally speaking, specific values and data (such as percentages, standard errors, p-values, etc.) should not be included in the abstract. Rather, this part of the abstract should provide readers with an overview.”* [*https://www.cwauthors.com/article/what-to-include-and-exclude-in-an-abstract*](https://www.cwauthors.com/article/what-to-include-and-exclude-in-an-abstract)

*Moreover, most importantly, emphasizing a p-value in an abstract is inconsistent with best practices vis JAMA: see Halabi, S., & Day, S. (2019). Improved Reporting in Abstracts When Uncertainty Is Inevitable. JAMA Network Open, 2(12), e1917543-e1917543.*

*We feel that reporting a p-value in the abstract distracts from the more complicated picture of findings we obtained. However, we remain open to the editor’s guidance on this matter.*

2. Sample size/power: It was quite strange to find no sample size/power justification, considering the matched design, using the primary response variable. Please also mention the statistical test, 1-, or 2-sided, 5% level of significance, and desired effect size. Note, the manuscript is submitted as a "Clinical Trial", where, sample size/power statements are essential. Create a separate subsection

*We think it’s important to point out -- as is noted in the paper on page 6 -- “The study was not originally conceived of as a formal clinical trial, but was later registered as a clinical trial at the request of the PLOS ONE editors (ID: NCT06014970).” We would also note that we have provided extensive detail on sample size in the manuscript or supplement (e.g., detailed cell sizes), each analysis has degrees of freedom reported, and estimates of effect size. We concur we did not include our power analyses in the draft. To improve clarity, we have added this information to our data analysis section (p. 6-7):*

*“With (n= 461) intervention students (n= 420) in matched comparison schools, the study is well powered to identify all repeated measures, between, and between -within interactions. Power analysis indicated sufficient power to detect medium to large effects with power estimates well above .95. Power analysis using g power indicated that the power to detect small effects for the most complicated analyses (using effect size f = .1, and desired power = .95, alpha = .050, two tailed) would require 912 denominator degrees of freedom and total n = 464. Power to detect small effects with desired power = .80 (again alpha = .050, two tailed) would require 608 denominator degrees of freedom and total n = 312. The above suggests all analyses were power above .80 with most in the above .90-.95 range.”*

*The following citations were added:*

*Faul, F., Erdfelder, E., Buchner, A., & Lang, A.-G. (2009). Statistical power analyzes using G*Power 3.1: Tests for correlation and regression analyses.* Behavior Research Methods *,* 41 *, 1149-1160.*

*Faul, F., Erdfelder, E., Lang, A.-G., & Buchner, A. (2007). G*Power 3: A flexible statistical power analysis program for the social, behavioral, and biomedical sciences.* Behavior Research Methods *,* 39 *, 175-191*

3. Statistical analysis: The proposed 3-way repeated measures factorial ANOVA is based wholesomely on Gaussian assumptions. There is no mention of the (nonparametric) alternatives to consider under violations to those assumptions, such as the Friedman's test. During analysis, Gaussianty checks should be performed.

*To our knowledge there is no non-parametric alternative to a 3-way repeated measures factorial ANOVA as there is for t-tests and ANOVA’s. However, we tested assumptions and reported on page 13 in the submitted draft “When Mauchly's test of sphericity indicated the assumption of sphericity had been violated and equal variances could not be assumed, the degrees of freedom for within-subjects effects were modified via the Greenhouse-Geisser procedure.”*

*To further clarify, we also specify that data were screened for non-normal distributions violations of skew and kurtosis. It was found that the BASC variable Attention displayed non-normal distributions (now more specifically described on p. 13); notably, the kurtosis value was mildly high (a value of 2.10). We have now added the additional clarification that post hoc tests were supplemented with tests that did not assume equal variances, specifying our use of the Friedman test. In the case of differences in significant findings between parametric and non-parametric alternatives, differences were described.* *(p. 13, Supplement p. 2).*

We thank you and the reviewers again for their thorough review and suggestions to improve the quality of the manuscript. We look forward to hearing from you.

Sincerely,

The Authors
